# Less restrictions in daily life: a clinical practice guideline for children with cancer

**DOI:** 10.1007/s00520-024-08537-9

**Published:** 2024-06-08

**Authors:** Debbie C. Stavleu, Renée L. Mulder, Demi M. Kruimer, Leontien C. M. Kremer, Wim J. E. Tissing, Erik A. H. Loeffen, On behalf of the social restrictions guideline panel, Laura R. Beek, Janneke H. P. Evers, Melanie M. Hagleitner, Daniëlle H. J. Martens, Jeroen G. Noordzij, Ida Ophorst, Janneke R. Ottens, Willemijn Plieger, Marjolijn S. W. Quaak, Tirza Schuerhoff, Judith Spijkerman, Alida F. W. van der Steeg, Marianne D. van de Wetering, Tom F. W. Wolfs

**Affiliations:** 1 University Medical Center Groningen, Beatrix Children’s Hospital, Department of Pediatric Oncology/Hematology, University of Groningen, Groningen, The Netherlands; 2 Princess Máxima Center for Pediatric Oncology, Utrecht, The Netherlands

**Keywords:** Pediatric oncology, Social restrictions, Clinical practice guideline

## Abstract

**Purpose:**

In current clinical practice, recommendations regarding restrictions in daily life for children with cancer are often lacking or not evidence-based. Critically reviewing the evidence and formulating recommendations are therefore of great importance as social restrictions (e.g., swimming, school attendance, sports) can impair the quality of life of these children severely. Therefore, our aim was to develop a clinical practice guideline for clinicians, children, and their parents regarding social restrictions in children with cancer.

**Methods:**

A comprehensive multidisciplinary panel was assembled, comprising 21 professionals and patient representatives. A systematic literature review was performed, including dual appraisal of all citations. The GRADE methodology was used to extract, summarize, and assess the evidence. Multiple in-person meetings were held to rank outcomes, discuss evidence, complete evidence-to-decision frameworks, and formulate recommendations. Final recommendations were unanimously supported by all panel members.

**Results:**

Six studies, including 758 children, formed the evidence base for the recommendations. Given the scarcity of the available evidence and various designs of studies in children with cancer, additional evidence was extracted from adult oncology guidelines, and shared expert opinions were utilized. In total, 14 recommendations were formulated of which multiple result in changes in current policy and standard of practice in the Netherlands. Topics covered in this guideline are swimming, having pets, visiting the zoo or farm, performing sports or high-velocity events, attending school or kindergarten, and use of public transport. This guideline is not intended to provide recommendations for patients after end of treatment, for palliative care settings, or for children undergoing a stem cell transplantation.

**Conclusions:**

In this clinical practice guideline, we provide recommendations regarding restrictions in daily life in children with cancer. These include evidence-based recommendations and, in the absence of sufficient evidence, recommendations based on expert evidence. With these recommendations, we provide guidance for clinicians, children, and parents and contribute to improving quality of life for children with cancer.

**Supplementary Information:**

The online version contains supplementary material available at 10.1007/s00520-024-08537-9.

## Introduction

Improving quality of life has become increasingly important in care for children with cancer. Due to improved survival rates, there is an increased focus on morbidity and adverse effects of anticancer treatment [1, 2]. To prevent adverse health problems, such as infections and bleeding, social restrictions have been defined for children with cancer related to school attendance, traveling on public transport, pets, hygiene measures, and swimming [3]. However, these social restrictions can potentially impair the quality of life of these children severely [4, 5].

Within the Netherlands, there is large variation in current supportive care practices, including social restrictions [6]. The majority of these recommendations regarding social restrictions for children with cancer are not evidence-based. Activities such as school attendance, swimming, visiting crowded places, or performing sports are restricted without justified or well-founded reasoning—maybe even unnecessarily, with potentially detrimental effects on quality of life.

Thus, critically reviewing and assessing the available evidence to formulate recommendations is of great importance. Guidance is necessary in order to provide the best possible care for these children, balancing cautiousness and restrictiveness.

Therefore, our aim was to develop a clinical practice guideline (CPG) regarding social restrictions in children with cancer by first establishing an overview of the available evidence and subsequently formulating recommendations for clinicians, children, and their parents. We explicitly aimed to provide recommendations even in absence of evidence, to establish clinical consensus and provide clinicians with a comprehensive guideline.

## Methods

### Guideline panel

A national, comprehensive multidisciplinary panel was assembled, comprising 21 professionals and patient representatives from the Netherlands. The panel included pediatric oncologists, pediatricians, a children’s psychologist, a child life specialist, a surgeon, a pediatric infectious disease specialist, a patient representative, nurse specialists, guideline specialists, and several researchers (see Supplemental Materials S1). Members were invited on the basis of their experience and knowledge on the topic. Moreover, the patient and parent representative organization was involved, to make it as applicable, clear, and usable for the patients and parents as possible. The core group (DS, RM, DK, LK, WT, EL) provided all the preparatory documents including methodology, study details, and results.

Between 2020 and 2022, multiple in-person panel meetings were held to rank outcomes, discuss evidence, and formulate recommendations.

### Guideline scope

With this guideline, our aim was to formulate recommendations regarding social restrictions in children with cancer aged 0–18 years. In addition, we explicitly aimed to provide recommendations even in absence of evidence, in order to provide recommendations for consistent and evidence-based clinical practice.

All recommendations are aimed at children with cancer receiving anticancer treatment with curative intent. These recommendations apply for out-patient settings, not for hospitalized patients. This guideline is not intended to provide recommendations for patients after end of treatment, for palliative care settings, or for children undergoing a stem cell transplantation.

It was attempted to make recommendations as general as possible and applicable for everyone. However, some recommendations may not apply or should be adjusted for the readers’ specific region or country.

### Existing guidelines and clinical questions

Existing international guidelines on social restrictions published until November 2019 were searched (GIN [7], NICE [8], IPOG [9], ASCO [10]) and evaluated for the applicability and completeness of these guidelines. In the absence of an applicable evidence-based guideline for children with cancer, clinical questions were defined by the core group. An overview of all clinical questions is shown in Supplemental Materials S2.

### Search strategy and selection criteria

An extensive systematic literature search (see Supplemental Materials S3) was performed in collaboration with a medical librarian. We searched the electronic databases PubMed, Embase, Cochrane CENTRAL, and CINAHL.

Inclusion and exclusion criteria were defined by the core group. Importantly, all children with cancer aged 0 to 18 years were included. Studies should have investigated any kind of social restriction. We only included controlled studies, applying a two-step approach by first including randomized controlled trials (RCTs) and in case of insufficient or inconclusive evidence other controlled and observational studies. Studies that only included children who had already undergone a stem cell transplantation were excluded, as we considered this a non-representative population.

It was agreed that when not enough studies were identified (*n* < 5 per topic), we extrapolated from evidence-based guidelines in other pediatric patient populations (e.g., infectious diseases, hematology) or guidelines in adult oncology patients (applicability depending on clinical question).

### Evidence selection and quality assessment

Study identification was performed independently by two reviewers. Initially, titles and abstracts were screened, followed by full-text assessment. Discrepancies were resolved by consensus after discussion between the two reviewers and a third, independent reviewer (EL).

Detailed information from each eligible study was extracted into evidence tables. The methodological quality of each single study was assessed and scored for risk of bias. For RCTs, the Risk of Bias tool v2 from the Cochrane handbook was used [11]. For non-RCT studies, we combined the risk of bias criteria for observational studies, as described in the Handbook of the International Guideline Harmonization Group [12], with specific aspects of the Cochrane RCT tool [11]. By combining these tools, we aimed to have the best possible tool to assess the risk of bias in our types of studies. These risk of bias assessment criteria for non-RCT studies are shown in Supplemental Materials S4.

All evidence was outlined in summary of findings’ tables. The quality of the total body of evidence was assessed by the Grading of Recommendations Assessment, Development and Evaluation (GRADE) approach [13, 14]. The data extraction, risk of bias assessment, and GRADE assessment were independently performed by two reviewers (DS, DK). Discrepancies were resolved by consensus or a third reviewer (EL).

### Translating evidence into recommendations using the evidence-to-decision framework

The GRADE evidence-to-decision framework was used to translate evidence into recommendations [14]. Within this framework, for every clinical question the benefits and harms, resource use, equity, acceptability, and feasibility were discussed and recommendations were formulated by the guideline panel. If no studies were identified, we carefully considered expert consensus (expert opinion). Final recommendations had to be unanimously supported by all panel members.

The GRADE terminology for evidence-based guidelines was used, such as “we suggest” or “we recommend” [13]. For the expert-based recommendations, the terminology from a recent paper published by the international Pediatric Oncology Guidelines in supportive care (iPOG) Network [15] was applied. The wording “we believe” was used to emphasize that these recommendations are based on expert opinion and group consensus.

We also formulated good practice statements [16] for recommendations that were considered a part of good clinical practice, but are not specifically studied (because this is not achievable or not deemed necessary).

Within the overview of all recommendations (Table 2), a color coding system was used to improve understandability and to emphasize the strength of the recommendations.

## Results

**Fig. 1:**
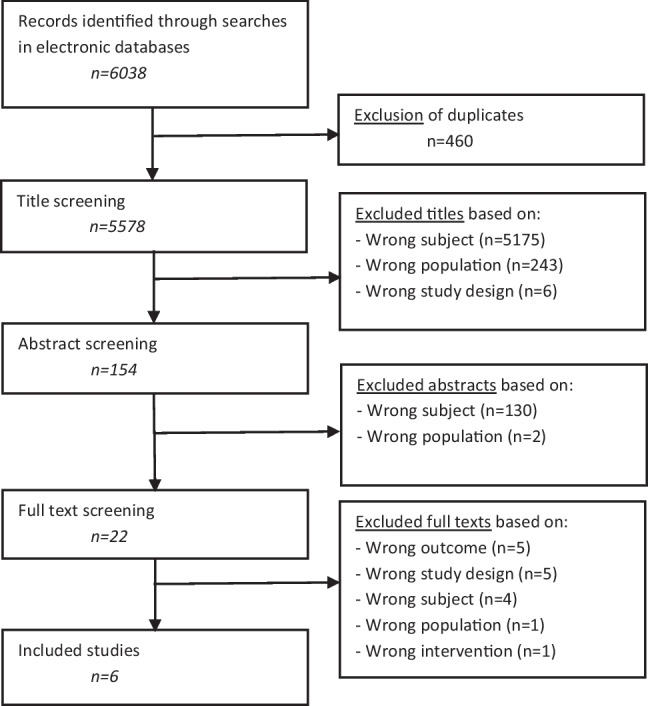
Flow diagram study selection

In total, 6038 unique citations were identified in initial literature search (September 2019) and two update searches (latest: February 2023). Six primary studies (2 RCTs, 2 retrospective cohort studies, 1 pre- and post- intervention study, 1 case–control study) were included with a total number of 758 participants (see Fig. 1). All primary study characteristics are shown in Supplemental Materials S5.

An overview of the included studies, the evidence tables, and the GRADE assessments can be found in Supplemental Materials S6–7. In Table 1, the conclusions of evidence of the included studies are presented. In Table 2, a list of all recommendations is shown.

**Table 1:** Conclusions of evidence related to social restrictions in children with cancer

Conclusion of evidence	Quality of evidence
Bath toy use	
Significantly more bath toy use in group infected with *Pseudomonas* compared to the group without *Pseudomonas* infection	⨁◯◯◯ (1 study (17)) **VERY LOW** quality of evidence
Bubble bath use	
Significantly more bubble bath use in group infected with *Pseudomonas* compared to the group without *Pseudomonas* infection	⨁◯◯◯ (1 study (17)) **VERY LOW** quality of evidence
Chlorhexidine use	
No significant differences in prevalence of infections were seen in the experimental bath wipes group versus the standard bath wipes group	⨁⨁◯◯ (1 study (21)) **LOW** quality of evidence
Overall, no significant differences in prevalence of infections between patients with vs. without chlorhexidine bathing Significantly lower prevalence of infections in patients with vs. without chlorhexidine bathing in specific age group 12–21 years	⨁◯◯◯ (1 study (18)) **VERY LOW** quality of evidence
No significant differences in prevalence of infections were seen in the chlorhexidine bathing group versus the control group	⨁⨁◯◯ (1 study (19)) **LOW** quality of evidence
Pets	
Restriction of pets at home was not significantly associated with a decreased risk of any type of infection	⨁◯◯◯ (1 study (4)) **VERY LOW** quality of evidence
Social restrictions	
Restriction of social contact was not significantly associated with a decreased risk of any type of infection	⨁◯◯◯ (1 study (4)) **VERY LOW** quality of evidence
Swimming	
No significant difference in prevalence of infections in the swimmer group versus the non-swimmer group No significant difference in prevalence of infections in the frequent swimmer group versus the infrequent/non-swimmer group	⨁◯◯◯ (1 study (22)) **VERY LOW** quality of evidence

**Table 2:**
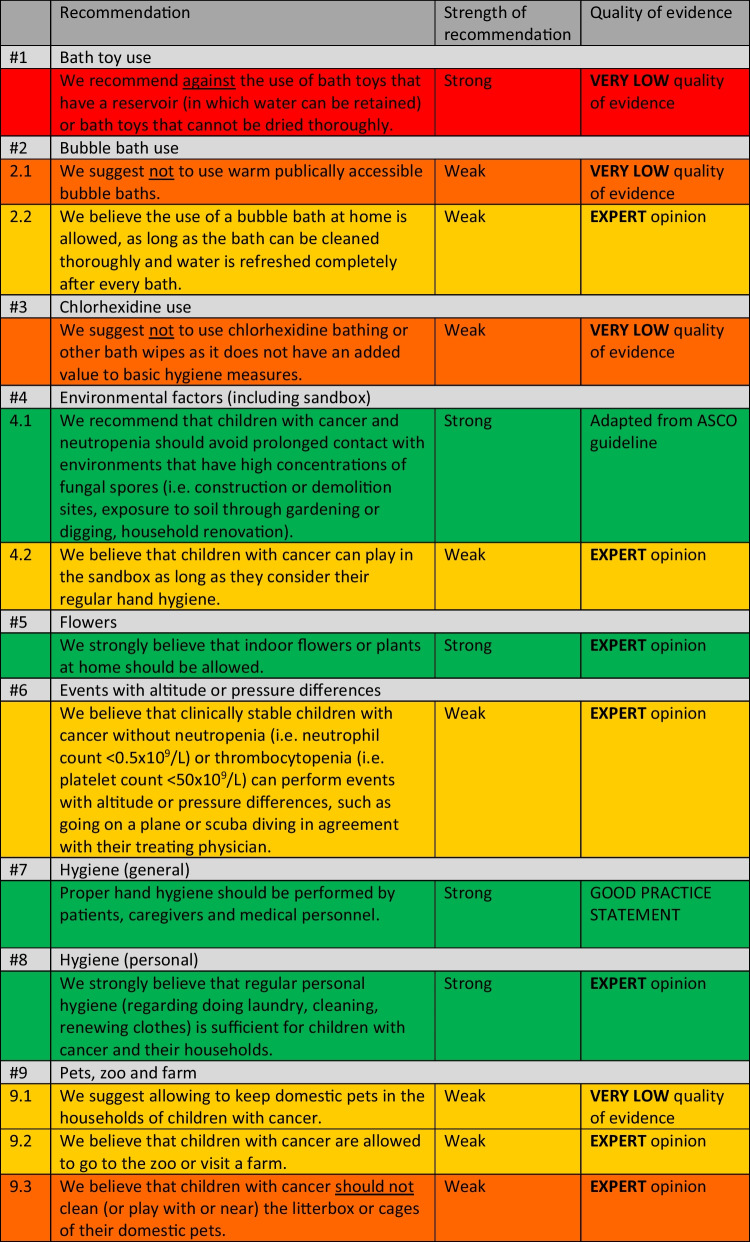

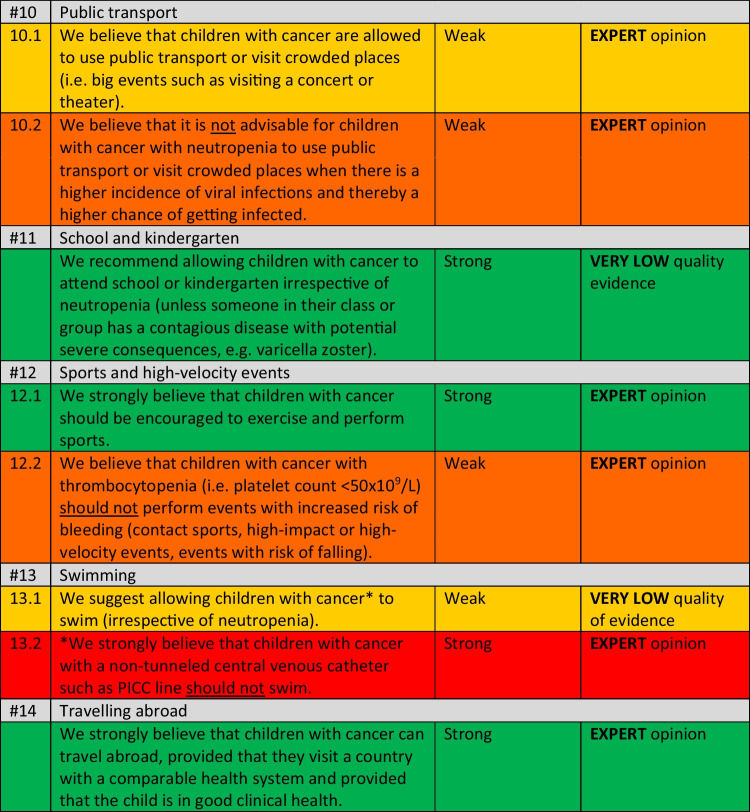
Overview of social restriction recommendations for children with cancer

*The color coding in this table emphasizes the strength of the recommendation and shows if something is advised (green (strong) or yellow (moderate)) or discouraged (orange (moderate) or red (strong))

All recommendations and their evidence-to-decision processes are discussed per subject. Given the extent of all recommendations, only conclusions and important considerations of the guideline panel are shown. Full details are shown in Supplemental Materials S7.

### Bath toy use

#### Recommendation 1

We recommend against the use of bath toys that have a reservoir (in which water can be retained) or bath toys that cannot be dried thoroughly (STRONG recommendation, VERY LOW quality of evidence).

#### Evidence to decision

One case–control study [17] in children with cancer was identified. In this study [17], significantly more bath toy use was reported in the group infected with *Pseudomonas aeruginosa* compared to the group without *Pseudomonas aeruginosa* infection.

The guideline panel agrees that bath toys with a reservoir in which water can be retained should not be used in children with cancer. The still standing water in the reservoir, for example, in the inside of a bath toy as in the included study, is a reservoir for several bacteria like *P. aeruginosa*, which can cause severe infections in these children. Also, toys that cannot be dried thoroughly are prone to colonization with bacteria and should therefore not be used.

Despite the very low quality of evidence, the panel decided to formulate a *strong* recommendation because of the expert opinions about the infectious risks.

It is not necessary to dispose all bath toys for (younger) children with cancer during their treatment. The panel agrees that if toys can be dried thoroughly and if there is no reservoir in which water can be retained, the toys are probably not an infectious risk and can be used safely. Note that this also accounts for sponges, towels, and other items that become wet during showering or bathing.

### Bubble bath use

#### Recommendation 2.1

We suggest not to use warm publically accessible bubble baths (WEAK recommendation, VERY LOW quality of evidence).

#### Recommendation 2.2

We believe the use of a bubble bath at home is allowed, as long as the bath can be cleaned thoroughly and water is refreshed completely after every bath (WEAK recommendation, EXPERT opinion).

#### Evidence to decision

One case–control study [17] in children with cancer was identified. In this study [17], significantly more bubble bath use was reported in the group infected with *Pseudomonas* compared to the group without *Pseudomonas* infection.

The guideline panel believes the infectious risk in public bubble baths is relatively high because of the amount of people that enter the bubble baths, the constant high temperature of the bubble baths that form a good growth environment for bacteria, and most importantly, the fact that, for these publically accessible bubble baths, water is not frequently refreshed.

However, the guideline panel believes that if a private bubble bath can be cleaned properly before the use of the bath and water can be completely refreshed, the use of a bubble bath at home (or at a vacation accommodation) is allowed.

### Chlorhexidine use

#### Recommendation 3

We suggest not to use chlorhexidine bathing or other bath wipes as it does not seem to have an added value to basic hygiene measures (WEAK recommendation, VERY LOW quality of evidence).

#### Evidence to decision

Two studies in children with cancer [18, 19] show inconsistent results regarding chlorhexidine bathing. Although one RCT [19] reported no overall significant differences in the prevalence of infections between patients with vs. without chlorhexidine bathing, there was a significantly higher rate of central line-related blood stream infection (CLABSI) in the chlorhexidine group aged 12–21 years. However, the validity of this outcome is difficult to assess due to several reasons (i.e., age groups not pre-defined, regular basic hygiene measures probably confounding). A non-randomized pre- and post-intervention study [18] showed no significant differences in prevalence of infections in the chlorhexidine bathing group versus the control group.

Also, a third study [20] on the use of chlorhexidine bath wipes showed no significant differences in prevalence of infections.

With the current evidence, the guideline panel does not see any added value for chlorhexidine bathing, and we consider it more of a burden to these children. Therefore, the panel suggests not to use chlorhexidine bathing as it does not seem to have an added value to basic hygiene measures.

### Environmental factors (including sandbox)

#### Recommendation 4.1

We recommend that children with cancer and neutropenia should avoid prolonged contact with environments that have high concentrations of fungal spores (i.e., construction or demolition sites, exposure to soil through gardening or digging, household renovation) (STRONG recommendation, ASCO and IDSA guideline [21]).

#### Recommendation 4.2

We believe that children with cancer can play in the sandbox as long as they consider their regular hand hygiene (WEAK recommendation, EXPERT opinion).

#### Evidence to decision

No evidence in children with cancer was identified. However, a recommendation by the ASCO and IDSA [21] guideline was used for the decision by the guideline panel. The guideline panel strongly agreed that the stated environmental sites [21] indeed could contain high levels of fungal spores and could therefore be a potential danger. Although this recommendation was not specifically made for children, we believe that it is also applicable to them.

The guideline panel specifically made a recommendation about playing in the sandbox, as this is a clinically relevant subject for parents and children. No evidence in pediatric oncology patients or other guidelines were identified. The guideline panel believes that children with cancer should be allowed to play in the sandbox, either at home, at the playground, or at school, as long as they consider their regular hand hygiene.

### Flowers

#### Recommendation 5

We strongly believe that indoor flowers or plants at home should be allowed (STRONG recommendation, EXPERT opinion).

#### Evidence to decision

No evidence in children with cancer was identified.

The guideline panel believes that indoor flowers and plants at home should be allowed. We believe the risk of infection of just having plants or flowers in the house is very minimal. The panel does suggest additional hygiene measures, such as refreshing the water of the flowers often, and proposes that the children do not play with or help cleaning the soil of the plants.

### Events with altitude or pressure differences

#### Recommendation 6

We believe that clinically stable children with cancer without neutropenia (i.e., neutrophil count < 0.5 × 10^9^/L) or thrombocytopenia (i.e., platelet count < 50 × 10^9^/L) can perform events with altitude or pressure differences, such as going on a plane or scuba diving in agreement with their treating physician (WEAK recommendation, EXPERT opinion).

#### Evidence to decision

No evidence in children with cancer was identified.

The guideline panel believes that children in a stable phase of their treatment without severe neutropenia or thrombocytopenia should be allowed to perform these events, in accordance with their treating physician.

### Hygiene (general)

#### Recommendation 7

Proper hand hygiene should be performed by patients, caregivers, and medical personnel (STRONG recommendation, GOOD PRACTICE STATEMENT).

#### Evidence to decision

The recommendation from the ASCO and IDSA [21] guideline was used, and expert opinions were discussed. The guideline panel strongly agrees that proper hand hygiene in concordance with local protocols is very important for patients, caregivers, and medical personnel. We therefore formulated a recommendation in line with the recommendation from the ASCO and IDSA guideline.

### Hygiene (personal)

#### Recommendation 8

We strongly believe that regular personal hygiene (regarding doing laundry, cleaning, renewing clothes) is sufficient for children with cancer and their households (STRONG recommendation, EXPERT opinion).

#### Evidence to decision

No evidence in children with cancer was identified.

The guideline panel agrees that basic hygiene measures are sufficient for children with cancer. We believe that as long as the household is cleaned in a normal way, this is sufficient. There is no need to intensify (in frequency or in use of extra cleaning products) any of these personal hygiene measures such as cleaning the house or doing laundry.

### Pets, zoo, and farm

#### Recommendation 9.1

We suggest allowing to keep domestic pets in the households of children with cancer (WEAK recommendation, VERY LOW quality of evidence).

#### Recommendation 9.2

We believe that children with cancer are allowed to go to the zoo or visit a farm (WEAK recommendation, EXPERT opinion).

#### Recommendation 9.3

We believe that children with cancer should not clean (or play with or near) the litterbox or cage of their domestic pets (WEAK recommendation, EXPERT opinion).

#### Evidence to decision

One study (observational study) in children with cancer was included for this clinical question [4], in which restriction of pets at home was not significantly associated with a decreased risk of any type of infection. The guideline panel agreed that any restriction in pets at home is not necessary. If children consider their regular hand hygiene after playing with or touching their pet, we see no reason why any other form of restriction should be advised. We believe risk of infection from a pet is minimal, considering adequate hand hygiene, and that the quality of life would decrease if there would be any form of pet restriction.

We also believe that children with cancer should be allowed to visit the zoo or farm. If the children remain at distance from the animals, we anticipated no problems regarding infectious risks. If the children, for example, on a farm, touch the pets or feed them, they should again carefully consider their hand hygiene. However, we do suggest that children with cancer do not clean and play with or near the cages and/or litter boxes of the pets. We consider the infectious risk higher for these tasks, and it can easily—with no to minimal decrease in quality of life—be avoided by children with cancer.

Additionally, we also suggest that the pets of these children are regularly seen by a veterinarian and that they are in good health.

### Public transport

#### Recommendation 10.1

We believe that children with cancer are allowed to use public transport or visit crowded places (i.e., big events such as visiting a concert or theater) (WEAK recommendation, EXPERT opinion).

#### Recommendation 10.2

We believe that it is not advisable for children with cancer with neutropenia to use public transport or visit crowded places when there is a higher incidence of viral infections and thereby a higher chance of getting infected (WEAK recommendation, EXPERT opinion).

#### Evidence to decision

No evidence in children with cancer was identified.

The guideline panel agrees that there is no need to avoid public transport as long as basic hygiene measures such as hand hygiene are performed. Then, we believe the risk of infection remains minimal.

The guideline panel does feel that there is an exception for children with cancer and neutropenia, who should avoid the public transport or crowded places when there is a higher incidence of viral infections. In these months, there is a higher chance of getting infected. As the potential consequences of a viral infection can be big (for example, hospital admission because of fever, delay of chemotherapy, or the need for antiviral medication), we believe the public transport should be avoided when there is a higher incidence of viral infections.

### School and kindergarten

#### Recommendation 11

We recommend allowing children with cancer to attend school or kindergarten irrespective of neutropenia (unless someone in their class or group has a contagious disease with potential severe consequences, e.g., varicella zoster) (STRONG recommendation, VERY LOW quality evidence).

#### Evidence to decision

One study (observational study) in children with cancer was identified [4] which showed that restriction of social contact was not significantly associated with a decreased risk of any type of infection.

The guideline panel recognizes that the risk of infection at schools or kindergarten may be a concern to parents. However, we agree that going to school or kindergarten increases the quality of life of these children in such a way that it outweighs the harms of potential infections. Going to school is very important for the development of any child, also for children with cancer. It also has an important social aspect of seeing their friends and continuing with their life in the best possible way.

We strongly suggest that children stay at home when someone in their class or group has a contagious disease with potential severe consequences, e.g., varicella zoster. If this is the case, the guideline panel suggests that this will then be discussed by the treating physician for the specific patient to discuss the benefits and harms of going to school or kindergarten in that specific case.

### Sports and high-velocity events

#### Recommendation 12.1

We strongly believe that children with cancer should be encouraged to exercise and perform sports (STRONG recommendation, EXPERT opinion).

#### Recommendation 12.2

We believe that children with cancer with thrombocytopenia (i.e., platelet count < 50 × 10^9^/L) should not perform events with increased risk of bleeding (contact sports, high-impact or high-velocity events, events with risk of falling) (WEAK recommendation, EXPERT opinion).

#### Evidence to decision

No evidence in children with cancer was identified.

Firstly, the guideline panel strongly believes that children with cancer are allowed (and should be encouraged) to exercise and perform sports. It is always encouraged for children to perform sports and other physical activities. This greatly benefits their physical state and their quality of life.

However, the guideline panel feels that an exception needs to be made for children with thrombocytopenia (i.e., platelet count < 50 × 10^9^/L). In some types of activities, such as contact sports like boxing or rugby, high-impact or high-velocity events, and events with risk of falling, the risk of bleeding is too high when a child has thrombocytopenia. Therefore, these activities should be avoided in the event of thrombocytopenia. We suggest encouraging these children to perform activities that are safe, to ensure the positive effects of performing activities and sports.

### Swimming

#### Recommendation 13.1

We suggest allowing children with cancer* to swim (irrespective of neutropenia) (WEAK recommendation, VERY LOW quality of evidence).

#### Recommendation 13.2

*We strongly believe children with cancer with a non-tunneled central venous catheter such as PICC line should not swim (STRONG recommendation, EXPERT opinion).

#### Evidence to decision

In one retrospective cohort study [22], no significant difference in prevalence of infections in the swimmer group versus the non-swimmer group and in the frequent swimmer group versus the infrequent/non-swimmer group was reported. They report 34 infections in a total of 843 months (0.04% infection rate) in the swimmer group versus 13 infections in 506 months (0.025% infection rate), resulting in a risk ratio of 1.6 which they did not consider statistically significant (significance calculated based on 95% CI, but confidence intervals are not reported)[22].

Despite the lack of evidence, the guideline panel feels that an absolute restriction regarding swimming is not necessary. We believe not allowing the children to swim would decrease their quality of life. The panel judged the benefits (improving quality of life) to outweigh the harms (minimal risks both infectious and dislocation wise).

For children with an external tunneled central venous catheter, swimming is therefore allowed, provided that the insertion site and dressings can be cleaned and dried thoroughly and that there is an unwounded skin (i.e., no needle in the central venous access port) or sign of infection.

The guideline panel recognizes the fear for dislocation or problems with a central venous line from parents and children. Although not necessary, a suggestion is that the child can wear a wetsuit shirt (or a different type of tight shirt) so that the line is pushed against the body.

No studies investigated the risks of swimming in children with a non-tunneled line. The guideline panel believes that swimming with a non-tunneled line such as a peripheral inserted central catheter (PICC) line should not be allowed, given the increased infection risk for non-tunneled lines.

Regarding swimming location, the guideline panel believes that it should be possible to swim in all locations which are destined as swimming areas, for example, chlorinated water (including swimming lessons), the sea, or in open water, given that there is no general advice against this from the local authorities.

### Traveling abroad

#### Recommendation 14

We strongly believe that children with cancer can travel abroad, provided that they visit a country with a comparable healthcare system and provided that the child is in good clinical health (STRONG recommendation, EXPERT opinion).

#### Evidence to decision

No evidence in children with cancer was identified.

The guideline panel believes that children with cancer can travel abroad, provided that they visit a country with a comparable healthcare system as their own and provided that the child is in good clinical health. Note, this should always be a careful consideration for the child as an individual, and therefore, this always needs to be discussed and allowed by the treating physician. It should not interfere with treatment and parents should carry a letter of the treating physician, in the event something happens when abroad.

## Discussion

In this clinical practice guideline, we provide evidence-based recommendations, expert-based recommendations, and best practice statements regarding social restrictions in children with cancer. These recommendations provide guidance for clinicians, children, and their parents and contribute to improving quality of life for children with cancer. As evidence-based recommendations for this area were lacking, this clinical practice guideline has the potential to greatly impact daily practice and therefore quality of care for children with cancer.

There is a major lack of evidence regarding the effects of social restrictions in children with cancer. We attempted multiple sensitive and broad literature searches, including other pediatric patient groups and adult oncology patients. Still the yield was low, and this is the most important limitation of this evidence-based guideline. In daily practice, healthcare providers and patients do not have the option to refrain from discussing options and making a decision about care. Therefore, the guideline panel agreed that we should go to great lengths to formulate recommendations. Therefore, the guideline panel provided recommendations based on expert opinions. This directly contributes to improving practice and should be implemented more often in guidelines. Nevertheless, clearly more research is needed in this niche.

A strength of this guideline is that it is, to the best of our knowledge, the first guideline regarding this (broad) topic that addresses all these (different) subjects that are important to children and their parents, both evidence-based and expert opinion-based. Also, it purposely attempted to formulate recommendations, even in absence of evidence, to not leave caregivers empty-handed. With that, we formulated insightful recommendations for important topics within daily clinical practice for children with cancer. A limitation, besides the scarcity of evidence as mentioned earlier, can be attributed to the evidence-to-decision framework. Certain important topics are discussed in this framework, but that could also mean that other topics are not addressed evenly. However, given the transparency of the EtD framework, it was attempted to fill it with as much information and considerations as possible, in order to make it as applicable as possible for other readers. It should also be noted that recommendations can be different per individual child per treatment per center, and this should always be considered by the treating physician when adapting the recommendations.

Then, shortly, we would like to address some barriers and facilitators. Note that these topics were not a part of the research but are addressed here because of its applicability, insight, and use for guideline readers. We consider the evidence-to-decision frameworks as a facilitator due to its transparency and thereby adaptation possibilities to local context; also, the variety in topics that are discussed in this guideline and the importance of these topics for patients are important facilitators. We consider the limited amount of evidence as the most important barrier in this guideline.

Throughout this process, it became clear how important current social restrictions are for children and their parents and how they affect their quality of life. This emphasizes the importance of the development of this guideline. Moreover, our process underlined the importance of including patient representatives and their perspectives and for increasing the knowledge and awareness for this subject.

Implementation of this evidence-based guideline can contribute to improving the quality of life of children with cancer. For example, we recommend that children with external central venous catheters are allowed to swim, which until now was discouraged in the Netherlands. This is an example of an important change in current practice in the Netherlands and an improvement in quality of life for these children. However, it remains important to always consider the benefits and harms for the individual child. This guideline can facilitate weighing these benefits and harms and balancing cautiousness and restrictiveness.

In conclusion, with effectuating this guideline, we aim to care and to contribute to improving the quality of life of children with cancer. These recommendations will play an important role in the daily lives of children with cancer and their parents, by establishing a balance between being cautious and thus protecting these vulnerable children for complications and participating in “normal” child life as much as possible.

### Supplementary Information

Below is the link to the electronic supplementary material.

Supplementary file1 (DOCX 274 KB)

## Declarations

### Disclaimer

The views expressed in the submitted article are those of the authors and guideline panel and might not reflect the official position of their institution.
