# Supplementary material for: Less restrictions in daily life: a clinical practice guideline for children with cancer
Source: Support Care Cancer. 2024 Jun 8;32(7):419. doi: 10.1007/s00520-024-08537-9 (PMC11162397; doi:10.1007/s00520-024-08537-9)
Supplement: Supplementary file 1 — Supplementary file1 (DOCX 274 KB) [file 520_2024_8537_MOESM1_ESM.docx]

**Supplemental Materials**

**S1: Members guideline panel…….…….…….…….…….…….…….…….…….…….…….…….…….…….…….…….…….…….…….…….…….…….…….…….…….…….2**

**S2: Clinical questions…….…….…….…….…….…….…….…….…….…….…….…….…….…….…….…….…….…….…….…….…….…….…….…….…….…………………3
S3: Complete literature search……………………………………………..…….…….…….…….…….…….…….…….…….…….…….…….…….…….…….…….…….……4-6
S4: Adjusted risk of bias assessment tool for non-RCT studies…….…….…….…….…….…….…….…….…….…….…….…….…….…….…….…….…….………7
S5: Study characteristics…….…….…….…….…….…….…….…….…….…….…….…….…….…….…….…….…….…….…….…….…….…….…….…….…….…………8-9**

**S6: Evidence table and GRADE assessment…….…….…….…….…….…….…….…….…….…….…….…….…….…….…….…….…….…….…….…….…….…..10-13
S7: Evidence-to-decision Frameworks…….…….…….…….…….…….…….…….…….…….…….…….…….…….…….…….…….…….…….…….…….…….…….14-66**

**Supplemental Materials S1: Members guideline panel**

| **Member** | **Affiliations** |
| --- | --- |
| D.C. Stavleu | MD, PhD student Princess Máxima Center for pediatric oncology, Utrecht and Beatrix Children’s Hospital (University Medical Center Groningen), Groningen |
| E.A.H. Loeffen | MD, PhD, pediatric resident, epidemiologist, postdoc researcher Princess Máxima Center for pediatric oncology, Utrecht and Beatrix Children’s Hospital (University Medical Center Groningen), Groningen |
| R.L. Mulder | PhD, postdoc researcher, guideline developer and methodologist Princess Máxima Center for pediatric oncology, Utrecht |
| D.M. Kruimer | MD, researcher Princess Máxima Center for pediatric oncology, Utrecht |
| L.C.M. Kremer | Pediatrician, MD, PhD, Prof. late effects in pediatric oncology, researcher Princess Máxima Center for pediatric oncology, Utrecht, University Medical Center Utrecht, Wilhelmina Children's Hospital, Utrecht, the Netherlands, Emma Children’s Hospital, Amsterdam UMC, University of Amsterdam, Amsterdam, the Netherlands |
| W.J.E. Tissing | Pediatric oncologist MD, PhD, Prof. Supportive Care, Princess Máxima Center for pediatric oncology, Utrecht and Beatrix Children’s Hospital (University Medical Center Groningen), Groningen |
| L.R. Beek | Psychologist, Princess Máxima Center for pediatric oncology, Utrecht |
| J.H.P. Evers | Nurse practitioner, Princess Máxima Center for pediatric oncology, Utrecht |
| M.M. Hagleitner | Pediatric oncologist, Princess Máxima Center for pediatric oncology, Utrecht |
| D.H.J. Martens | Pediatrician, Isala, Zwolle |
| J.G. Noordzij | Pediatrician, Reinier de Graaf Gasthuis, Delft |
| I. Ophorst | Pediatric oncology nurse, MSc, Princess Máxima Center for pediatric oncology, Utrecht |
| J. Ottens | Nurse practitioner, Beatrix Children’s Hospital (University Medical Center Groningen), Groningen |
| W. Plieger | Patient representative VKKN (Vereniging Kinderkanker Nederland) |
| M. Quaak | Pediatrician, MD, fellow pediatric infectious diseases and immunology, Sophia Children’s Hospital (Erasmus MC), Rotterdam |
| T. Schuerhoff | Child life specialist, Princess Máxima Center for pediatric oncology, Utrecht |
| J. Spijkerman | Pediatrician, MD, fellow pediatric oncology, Princess Máxima Center for pediatric oncology, Utrecht |
| M.D. van de Wetering | Pediatric oncologist, MD, PhD, Princess Máxima Center for pediatric oncology, Utrecht, SKION working group Supportive Care |
| T.F.W. Wolfs | Pediatric infectious disease specialist, MD, PhD, University Medical Center Utrecht, Wilhelmina Children's Hospital, Utrecht, the Netherlands |
| *A.F.W. van der Steeg* | *Pediatric surgeon, MD, Princess Máxima Center for pediatric oncology, Utrecht ** was involved only in the decision making on swimming* |

**Supplemental Materials S2: Clinical questions**

The purpose of this guideline is to provide recommendations for children with cancer, receiving anti-cancer treatment with **curative intent.** This accounts for all of the following patient groups. The guideline was not intended to provide recommendations for palliative care settings.

**PICO (1)**
*What is the effect of social restrictions (regarding risk of infections) on infections (prevalence and infectious complications) and other outcomes in children with any type of cancer and/or after stem-cell transplantation?*

P = Children (aged 0-18 years) with any type of cancer and/or after stem-cell transplantation
 receiving anti-cancer treatment with curative intent
I = Social restrictions regarding risk of infections (i.e. restriction in school attendance,
 kindergarten, visiting zoo or farm, pets, swimming (whirlpool, sauna visits), being in
 crowded places, public transport, intimacy, flowers, or other author defined social
 restrictions)
C = (No social restrictions)
O = Severe or mild infections (prevalence and infectious complications), quality of life,
 anti-cancer treatment-related complications (adjustments in therapy or delay), costs,
 mortality, event-free survival

**PICO (2)**
*What is the effect of social restrictions (regarding bleeding risk) on hemorrhagic complications and other outcomes in children with any type of cancer and/or after stem-cell transplantation?*

P = Children (aged 0-18 years) with any type of cancer and/or after stem-cell transplantation
I = Social restrictions regarding risk of bleeding (i.e. restriction in physical contact sports,
 high-velocity sports, high impact or high energetic sports, sports with high risk of falling
 such as skiing or skating, playing with high risk of falling such as slides, high or low
 altitude events such as scuba diving or flying, rollercoaster rides or other author-defined
 social restrictions)
C = (No social restrictions)
O = Hemorrhagic complications (mild and severe), quality of life, anti-cancer treatment
 related complications, (adjustments in therapy or delay), costs, mortality, event-free
 survival

**PICO (3)**

*What is the effect of social restrictions, after a minor procedure or in patients with a venous access line with needle insertion, on infections and other outcomes in children with any type of cancer and/or after stem-cell transplantation?*

P = Children (aged 0-18 years) with any type of cancer and/or after stem-cell transplantation
 after a minor procedure (i.e. lumbar puncture) or with venous access line (central venous
 access ports) with needle insertion
I = Social restrictions (e.g. being in crowded places, showering, taking a bath, swimming)
C = (No social restrictions)
O = Severe or mild infections (prevalence and infectious complications), quality of life,
 anti-cancer treatment-related complications (adjustments in therapy or delay), costs,
 mortality, event-free survival

**PICO (4)**

*What is the effect of hygiene rules on infections and other outcomes in children with any type of cancer and/or after stem-cell transplantation?*

P = Children (aged 0-18 years) with any type of cancer and/or after stem-cell transplantation
I = Hygiene rules (e.g. cleaning, laundry, renewing of clothes, personal hygiene)
C = (No hygiene rules)
O = Severe or mild infections (prevalence and infectious complications), quality of life,
 anti-cancer treatment-related complications (adjustments in therapy or delay), costs,
 mortality, event-free survival

The guideline panel formed these four general clinical questions. In case of specific clinical questions of individual interventions further on in the process, these PICO’s will be divided into clinical sub-questions.

**S3: Complete literature search**

*Performed by Mw. H.W.J. Deurenberg*

ID Search

#1 (Cancer OR cancers OR cancer* OR oncology OR oncolog* OR neoplasm OR neoplasms OR neoplasm* OR carcinoma OR carcinom* OR tumor OR tumour OR tumor* OR tumour* OR tumors OR tumours OR malignan* OR malignant OR hematooncological OR hemato oncological OR hemato-oncological OR hematologic neoplasms OR hematolo*):ti,ab,kw (Word variations have been searched)

#2 "P variant breed":ti

#3 MeSH descriptor: [Stem Cell Transplantation] explode all trees

#4 stem NEAR/2 cell NEAR/3 transplan*:ti,ab

#5 stem NEAR/2 cell NEAR/3 transplan*:kw

#6 [mh "bone marrow transplantation"]

#7 "bone marrow" NEAR/5 transplant*:ti,ab,kw

#8 "stem cell" NEAR/5 transplant*:ti,ab,kw

#9 #3 OR #4 OR #5 OR #6 OR #7 OR #8

#10 "P variant stam cel transplantatie".ti.

#11 MeSH descriptor: [Leukemia] explode all trees

#12 (leukemia or leukemi* or leukaemi*):ti,ab,kw

#13 (aml or anll or lymphoma or lymphom* or hodgkin* or T-cell or B-cell or non-hodgkin or sarcoma or sarcom* or Ewing* or osteosarcom* or wilms* or nephroblastom* or neuroblastom* or rhabdomyosarcom* or teratom* or hepatom* or hepatoblastom* or PNET or medulloblastom* or PNET* or (neuroectodermal adj2 tumors NEAR/2 primitive) or retinoblastoma or retinoblastom* or meningiom* or gliom*):ti,ab,kw

#14 [mh "lymphatic vessel tumors"]

#15 MeSH descriptor: [Lymphatic Vessel Tumors] explode all trees

#16 [mh lymphoma] OR [mh "neoplasms, complex and mixed"] OR [mh "neoplasms, connective and soft tissue"] OR [mh "neoplasms, germ cell and embryonal"] OR [mh "neoplasms, glandular and epithelial"] OR [mh “neoplasms, gonadal tissue”] OR [mh “neoplasms, nerve tissue”] OR [mh “neoplasms, plasma cell”] OR [mh “neoplasms, vascular tissue"] OR [mh “neoplasms by site"] OR [mh “neoplasms, hormone-dependent"] OR [mh “neoplasms, radiation-induced"] OR [mh “neoplastic syndromes, hereditary”]

#17 ((brain NEAR/1 tumor*) OR (brain NEAR/1 tumour) OR (brain NEAR/1 neoplasm*) or (central NEAR/1 nervous NEAR/1 system NEAR/1 neoplasm*) OR (central NEAR/1 nervous NEAR/1 system NEAR/1 tumo*) or (central NEAR/1 nervous NEAR/1 system NEAR/1 cancer*) or (brain NEAR/1 cancer*) or (brain NEAR/1 neoplasm*) or (intracranial NEAR/1 neoplasm*) or (leukemia NEAR/1 lymphocytic NEAR/1 acute*)):ti,ab,kw

#18 #11 OR #12 OR #13 OR #14 OR #15 OR #16 OR #17=P leukemia

#19 #1 OR #9 OR #18

#20 ((pediatric NEAR/3 oncolog*) OR (paediatric NEAR/3 oncol*) OR (child* NEAR/3 (cancer* OR tumor* OR tumour* OR neoplasm*))):ti,ab,kw

#21 [mh "young adult"] OR [mh child] OR [mh infant]

#22 ((young NEAR/1 adult*) OR child* OR infant* OR pediatr* OR paediatr* OR perinat* OR neonat* OR newborn* OR infan* OR boy OR boys OR girl OR girls OR kid OR kids or schoolage* or juvenil* or teenage* or adolescen* or toddler*):ti,ab,kw

#23 #20 OR #21 OR #22

#24 #18 AND #23

#25 [mh "Platelet Transfusion"]

#26 [mh Plateletpheresis]

#27 [mh "Blood Platelets"]

#28 ((platelet* OR thrombocyte*) NEAR/5 (prophyla* OR transfus* OR infus* OR administ* OR requir* OR need* OR product* OR component* OR concentrate* OR apheres* OR pooled OR single NEAR/1 donor OR random NEAR/1donor)):ti,ab,kw

#29 (thrombocytopheres* or plateletpheres*):ti,ab,kw

#30 #25 OR #26 OR #27 OR #28 OR #29

#31 [mh "blood component transfusion"] OR [mh "erythrocyte transfusion"]

#32 ((blood NEAR/3 transfus*) or (erythrocyt* NEAR/2 transfus*)):ti,ab,kw

#33 ((erythrocy* OR hemoglobin* OR haemoglobin*) NEAR/5 (prophyla* OR transfus* OR infus* OR administ* OR requir* OR need* OR product* OR component* OR concentrate* OR apheres* OR pooled OR single NEAR/1 donor OR random NEAR/1donor)):ti,ab,kw

#34 #31 OR #32 OR #33

#35 [mh "blood component transfusion"] OR [mh "erythrocyte transfusion"] OR [mh "platelet transfusion"]

#36 [mh "Platelet Count"]

#37 #35 OR #36

#38 #30 OR #34 OR #37

#39 #24 AND #38

#40 (#19 or #20) AND (#21 or #22)

#41 [mh "Social Participation"]

#42 (social NEAR/3 (participat* OR contact OR contacts OR reintegrat*)):ti,ab,kw

#43 (activity NEAR/2 limitat*):ti,ab,kw

#44 [mh "Social Isolation"]

#45 [mh "Interpersonal Relations"]

#46 social NEAR/2 (restriction* OR interaction* OR competenc* OR participat* OR contact* OR reintegrat*):ti,ab,kw

#47 (restrict* NEAR/2 participati* NEAR/5 (activities or relations*)):ti,ab,kw

#48 [mh hygiene] OR [mh "hand hygiene"] OR [mh "skin care"]

#49 showering OR bath* OR hygiene OR cleani* OR laundry OR (renew* NEAR/2 cloth*):ti,ab,kw

#50 [mh Pets]

#51 ((school NEAR/3 attendan*) OR (public NEAR/2 transpor*) or bus or train):ti,ab,kw

#52 [mh "Animals, Zoo"]

#53 [mh "schools, nursery"]

#54 [mh Nurseries] OR [mh "Child Day Care Centers"]

#55 (child* NEAR/2 day NEAR/2 care NEAR/3 center):ti,ab,kw

#56 [mh "Swimming Pools"]

#57 [mh Swimming] OR [mh boxing] OR [mh skating] OR [mh soccer] OR [mh hockey] OR [mh diving] OR [mh "weight lifting"] or [mh wrestling] OR [mh "youth sports"]

#58 (boxing OR skat* OR soccer OR diving OR (weight NEAR/1 lift*) OR wrestling OR swimming OR (scuba NEAR/2 diving) OR flying OR rollercoast* OR football* or hockey):ti,ab,kw

#59 #41 OR #42 OR #43 OR #44 OR #45 OR #46 OR #47 OR #48 OR #49 OR #50 OR #51 OR #52 OR #53 OR #54 OR #55 OR #56 OR #57 OR #58

#60 #40 AND #59

**Cochrane search**

P= children with cancer = set 18 AND set 23

1. cancer = set 18

2. age = set 23

3. social restrictions  =set 59

4. result = set 60 = P children with cancer + social restrictions

Database: Ovid MEDLINE(R) ALL <1946 to December 11, 2020>

Search Strategy:

--------------------------------------------------------------------------------

1 "coc social restrictions".ti. (0)

2 exp stem cell transplantation/ or exp hematopoietic stem cell transplantation/ (83929)

3 (stem adj2 cell adj3 transplan*).tw. (50727)

4 (stem adj2 cell adj3 transplan*).kf. (7551)

5 bone marrow transplantation/ (44746)

6 ("bone marrow" adj5 transplant$).tw. (38419)

7 ("bone marrow" adj5 transplant$).kf. (1865)

8 ("stem cell" adj5 transplant$).tw. (51747)

9 ("stem cell" adj5 transplant$).kf. (7713)

10 or/2-9 (151154)

11 exp Leukemia/ (234051)

12 (leukemia or leukemi* or leukaemi*).tw. (269738)

13 (leukemia or leukemi* or leukaemi*).kf. (32354)

14 (aml or anll or lymphoma or lymphom* or hodgkin* or T-cell or B-cell or non-hodgkin or sarcoma or sarcom* or Ewing* or osteosarcom* or wilms* or nephroblastom* or neuroblastom* or rhabdomyosarcom* or teratom* or hepatom* or hepatoblastom* or PNET or medulloblastom* or PNET* or (neuroectodermal adj2 tumors adj2 primitive) or retinoblastoma or retinoblastom* or meningiom* or gliom*).tw. (834591)

15 (aml or anll or lymphoma or lymphom* or hodgkin* or T-cell or B-cell or non-hodgkin or sarcoma or sarcom* or Ewing* or osteosarcom* or wilms* or nephroblastom* or neuroblastom* or rhabdomyosarcom* or teratom* or hepatom* or hepatoblastom* or PNET or medulloblastom* or PNET* or (neuroectodermal adj2 tumors adj2 primitive) or retinoblastoma or retinoblastom* or meningiom* or gliom*).kf. (90826)

16 exp lymphatic vessel tumors/ or exp lymphoma/ or exp "neoplasms, complex and mixed"/ or exp "neoplasms, connective and soft tissue"/ or exp "neoplasms, germ cell and embryonal"/ or exp "neoplasms, glandular and epithelial"/ or exp neoplasms, gonadal tissue/ or exp neoplasms, nerve tissue/ or exp neoplasms, plasma cell/ or exp neoplasms, vascular tissue/ or exp neoplasms by site/ or exp neoplasms, hormone-dependent/ or exp neoplasms, radiation-induced/ or exp neoplastic syndromes, hereditary/ (2727316)

17 ((brain adj tumo?r*) or (brain adj neoplasm?) or (central adj nervous adj system adj neoplasm?) or (central adj nervous adj system adj tumo?r?) or (central adj nervous adj system adj cancer?) or (brain adj cancer*) or (brain adj neoplasm*) or (intracranial adj neoplasm*) or (leukemia adj lymphocytic adj acute*)).tw. (49779)

18 ((brain adj tumo?r*) or (brain adj neoplasm?) or (central adj nervous adj system adj neoplasm?) or (central adj nervous adj system adj tumo?r?) or (central adj nervous adj system adj cancer?) or (brain adj cancer*) or (brain adj neoplasm*) or (intracranial adj neoplasm*) or (leukemia adj lymphocytic adj acute*)).kf. (10686)

19 or/11-18 (3380178)

20 "variant neurocognitive P".ti. (0)

21 "P variant breed".ti. (0)

22 ((p?ediatric adj3 oncolog*) or (child* adj3 (cancer? or tumo?r? or neoplasm?))).tw. (39721)

23 ((p?ediatric adj3 oncolog*) or (child* adj3 (cancer? or tumo?r? or neoplasm?))).kf. (2540)

24 young adult/ or exp child/ or exp infant/ (3206941)

25 ((young adj adult?) or child??? or childhood or infant* or p?ediatr* or perinat* or neonat* or newborn* or infan* or boy? or girl? or kid? or schoolage* or juvenil* or teenage* or adolescen* or toddler?).tw. (2465097)

26 ((young adj adult?) or child??? or childhood or infant* or p?ediatr* or perinat* or neonat* or newborn* or infan* or boy? or girl? or kid? or schoolage* or juvenil* or teenage* or adolescen* or toddler?).kf. (336883)

27 (cancer* or oncolog* or neoplasm* or carcinom* or tumor* or tumour* or malignan* or hematooncological or hemato?oncological or hemato-oncological or (hematologic adj neoplasm*)).tw. (3361279)

28 (cancer* or oncolog* or neoplasm* or carcinom* or tumor* or tumour* or malignan* or hematooncological or hemato?oncological or hemato-oncological or (hematologic adj neoplasm*)).kf. (611739)

29 22 or 23 (40391)

30 10 or 19 or 29 (3481138)=stamcel transplantatie

31 24 or 25 or 26 (4126038)=kinderen

32 "zoekacties social restrictions en hygiene".ti. (0)

33 Social Participation/ (2558)

34 (social adj3 (participat* or contact? or reintegrat*)).tw. (10500)

35 (activity adj2 limitat*).tw. (3795)

36 Social Isolation/ (13929)

37 Interpersonal Relations/ (73010)

38 (social adj2 restriction?).tw. (649)

39 (restrict* adj2 participati* adj5 (activities or relations*)).tw. (141)

40 (interacti* adj3 social*).tw. (24227)

41 (social adj2 compet*).tw. (4008)

42 hygiene/ or hand hygiene/ or skin care/ (22978)

43 (showering or bath* or hygiene or cleani* or laundry or (renew* adj2 cloth*)).tw. (135913)

44 (showering or bath* or hygiene or cleani* or laundry or (renew* adj2 cloth*)).kf. (15536)

45 (social adj3 (participat* or contact? or reintegrat*)).kf. (756)

46 (activity adj2 limitat*).kf. (151)

47 (social adj2 restriction?).kf. (11)

48 (restrict* adj2 participati* adj5 (activities or relations*)).kf. (0)

49 (interacti* adj3 social*).kf. (1472)

50 (social adj2 compet*).kf. (276)

51 Pets/ (2641)

52 ((school adj3 attendan*) or (public adj2 transpor*) or bus* or train?).tw. (116944)

53 Animals, Zoo/ (5400)

54 schools, nursery/ (1474)

55 Nurseries/ (1087)

56 Child Day Care Centers/ (5063)

57 (child? adj2 day adj2 care adj3 center?).tw. (143)

58 (child? adj2 day adj2 care adj3 center?).kf. (21)

59 ((school adj3 attendan*) or (public adj2 transpor*) or buss* or train?).kf. (778)

60 exp Swimming Pools/ or exp Swimming/ (26392)

61 boxing/ or skating/ or soccer/ or hockey/ or diving/ or weight lifting/ or wrestling/ or youth sports/ (24034)

62 (boxing or skat* or soccer or diving or (weight adj lift*) or wrestling or swimming or (scuba adj2 diving) or flying or rollercoast* or football* or hockey*).tw. (69080)

63 (boxing or skat* or soccer or diving or (weight adj lift*) or wrestling or swimming or (scuba adj2 diving) or flying or rollercoast* or football* or hockey*).kf. (7172)

64 or/33-60 (432018)= standaard activiteiten met risico’s

65 61 or 62 or 63 (79457)= risicovolle activiteiten

66 hemorrhage/ or blood loss, surgical/ or exp oral hemorrhage/ or exp postoperative hemorrhage/ (105696)

67 (bleeding or (blood adj3 loss) or rebleeding or hemorrhage* or h?emostasis or complicat*).tw,kf. (1490880)

68 66 or 67 (1519709)=bloedverlies

69 30 and 31 and 64 (3539)

70 30 and 31 and 65 (150)

71 30 and 31 and 68 (39860)

72 exp Case Reports/ (2140140)

73 (case adj2 serie?).ti,ab,kf. (81166)

74 72 or 73 (2206004)

75 69 not 74 (3127)

76 70 not 74 (95)

77 (30 and 31 and (64 or 65)) not 74 (3200)=stamceltransplantatie + kinderen + activiteiten geen case reports

78 77 (3200)

79 limit 78 to yr="2019 -Current" (320)

**Medline search**

P= children with cancer = set 18 AND set 23

1. cancer = set 18

2. age restriction to children = set 23

3. social restrictions =set 64 or set 65

4. results = set 79 = set 77 and P children with cancer

**Supplemental Materials S4: Adjusted risk of bias assessment tool for non-RCT studies***For RCTs, the Risk of Bias tool v2 from the Cochrane handbook was used (11). For non-RCT studies, we combined the risk of bias criteria for observational studies, as described in the Handbook of the International Guideline Harmonization Group (12), with specific aspects of the Cochrane RCT tool (11). By combining these tools, we aimed to have the best possible tool to assess the risk of bias in our types of studies.*

| Selection bias | Is the study group representative? Cases and controls were selected based on comparable patient characteristics (i.e. age, gender and tumor type).  *Low risk if:* no significant differences between cases and controls with respect to age, gender and tumor type.  *High risk if:* cases and controls differ with respect to age, gender and tumor type (baseline imbalances caused by selection). |
| --- | --- |
|  |  |
| Attrition bias | Is complete outcome data for all the participants available in this study? Is the follow up adequate?   *Low risk if:* no missing data, reasons for missing data not related to outcome, missing data balanced across groups, proportion missing or plausible effect size nog enough to have a clinically relevant effect.  *High risk if:* imbalance in numbers or reasons, proportion missing or plausible effect size enough to have a clinically relevant effect, inappropriate use of imputation, ‘as treated’ analysis with substantial departure from allocation. |
|  |  |
| Detection bias | Are the outcome assessors blinded for important determinants related to the outcome?  *Low risk if:* the outcome assessors were blinded for important determinants related to the outcome  *High risk if:* no blinding or broken blinding ***and*** measurement likely to be influenced. |
|  |  |
| Reporting bias | Is the report complete? Are the outcomes that were planned to be measured also reported?  *High risk if:* Outcomes not reported as pre-specified or expected or outcomes reported incompletely so they cannot be entered in meta-analysis. |
|  |  |
| Confounding bias | Are the analyses adjusted for important confounding factors?  *Low risk if*: important prognostic factors (i.e. age, gender, diagnosis and risk stratification) were taken adequately into account. *High risk if:* important prognostic factors (i.e. age, gender, diagnosis and risk stratification) were inadequately or not taken into account. |
|  |  |
| Other bias | The following list of other potential sources of bias in a clinical study may aid detection of further problems;  *High if:*   - The conduct of the study is affected by interim results (e.g. recruiting additional participants from a subgroup showing more benefit). - There is deviation from the study protocol in a way that does not reflect clinical practice (e.g. *post hoc* stepping-up of doses to exaggerated levels). - There is pre-randomization administration of an intervention that could enhance or diminish the effect of a subsequent, randomized, intervention. - Inappropriate administration of an intervention (or co-intervention). - Contamination (e.g. participants pooling drugs). - Occurrence of ‘null bias’ due to interventions being insufficiently well delivered or overly wide inclusion criteria for participants (Woods 1995). - An insensitive instrument is used to measure outcomes (which can lead to under-estimation of both beneficial and harmful effects). - Selective reporting of subgroups. - Fraud. - Baseline imbalances for other reasons than through selection. - Other. |
|  |  |

**Supplemental Materials S5: Study characteristics**

| **Article  Author, year Study type** | **Population a. No. of patients b. Population** | **Case group*  a. Group definition**  **b. No. of patients, age, gender (% males)** | **Control Group  a. Group definition**  **b. No. of patients, age, gender (% males)** | **Included outcomes** | **Risk of bias assessment**  **a. Selection bias b. Attrition bias  c. Detection bias  d. Reporting bias e. Confounding bias f. Other bias** |
| --- | --- | --- | --- | --- | --- |
| Buttery et al, 1998 Case-control study | a. 32 patients b. Cases and matched controls (with cancer diagnoses) during Pseudomonas outbreak, single-center, 1997. | a. Patients with clinical infections during Pseudomonas outbreak (with the same strain, on the pediatric ward) b. 8 patients, mean age 4.5 years, 62.5% males | a. Inpatients on the pediatric ward during the Pseudomonas outbreak but *without* infections (2) b. 24 patients, mean age 8.3 years, 62.5% males | - Number of infections | a. High b. Low c. Low  d. Unclear e. High f. Low |
| Raulji et al, 2015 Pre- and post-intervention study | a. 330 patients b. All pediatric oncology inpatient admissions, single-center, 2008-2010. | a. Post-intervention group given daily sponge bathing with chlorhexidine  b. 140 patients; 49 patients aged 0-4, 35 patients aged 4-7, 19 patients aged 7-12, 37 patients aged 12-21; 60.7% males | a. Pre-intervention group of children *not* receiving chlorhexidine baths  b. 190 patients, 61 patients aged 0-4, 27 patients aged 4-7, 48 patients aged 7-12, 54 patients aged 12-21; 50.5% males | - Number of infections | a. High b. High c. Low  d. Unclear e. High f. High |
| Tramsen et al, 2016 Retrospective cohort study | a. 339 patients  b. Children with acute myeloid leukemia treated accordingly AML-BFM 2004, between 2004 and 2010 in Germany, Austria, Switzerland and the Czech Republic. | 37 hospitals completed a survey about anti-infective measures. The survey included questions about restriction in social contacts, pets at home, and food. (5) Restrictions were categorized as always restricted (2 points), restricted under certain circumstances (1 point) or never restricted (0 points). Therefore, higher numbers represent more restrictions. They gathered data about infectious complications and calculated an incidence rate ratio of infection per score. | | - Number of infections | a. Low b. Low c. Low d. Unclear  e. High f. Low |
| Robbins et al, 1999 Retrospective cohort study | a. 95 patients b. Pediatric oncology patients with a tunneled central venous catheter (CVC) in place (single- or double lumen), single-center, 1994-1996. | a. “Swimmers” (patients who swam at least once with catheter in place) b. 49 patients, median age 9 years, % males not provided. | a. “Non-swimmers”, (patients who did not swim at all) b. 46 patients, median age 8 years, % males not provided. | - Number of infections | a. Unclear b. High c. Low  d. Unclear e. High f. High |

| **Article  Author, year Study type** | **Population a. No. of patients b. Population** | **Case group*  a. Group definition  b. No. of patients, age, gender (% males)** | **Control Group  a. Group definition  b. No. of patients, age, gender (% males)** | **Included outcomes** | **RCT Risk of bias assessment**  **a. Selection bias (random sequence generation) b. Selection bias (allocation concealment) c. Performance bias d. Detection bias e. Attrition bias f. Reporting bias g. Other bias** |
| --- | --- | --- | --- | --- | --- |
| Kjellin et al, 2020 RCT | a. 127 patients b. Patients ≤21 years of age who were scheduled to undergo an autologous or allogeneic HCT. | a. Bath wipes, used once daily for 60 days post-HCT, containing allantoin, colloidal silver, preservatives, vitamin E, aloe, and lauryl glucoside.  b. 61 patients, median age 10 years (range 0.26-20.95 years), 27 males (44%). | a. Bath wipes, used once daily for 60 days post-HCT, contained rinse-free soap and lotion b. 66 patients, median age 6.1 years (range 0.43-21.12 years), 37 males (56%). | - Number of infections | a. Unclear b. Unclear c. High d. Low e. Low f. Unclear g. High |
| Zerr et al, 2020 RCT | a. 174 patients b. Pediatric oncology patients ≥2 months and <22 years who were receiving treatment or were undergoing allogeneic HCT, and had an eligible CVC (externally tunneled). | a. Patients underwent once-daily bathing for 90 days with either cloths impregnated with mild cleansers.  b. 88 patients, median age 5.5 years (range 2-12 years), 53 males (60,2%). | a. Patients underwent once-daily bathing for 90 days with 2% CHG-impregnated cloths.  b. 87 patients, median age 4 years (range 1-8 years), 51 males (58.6%). | - Number of infections - Adverse events | a. Low b. Low c. Low d. Unclear e. High f. Unclear g. Low |

**S6: Evidence table and GRADE assessment**

Buttery, 1998 – Bath toy use

| **Author, study design** | **No. of participants, total (cases vs controls) & Group definition** | **Results** | **Statistical methods** | **Effect size** | **Quality of evidence** |
| --- | --- | --- | --- | --- | --- |
| 1) Buttery, 1998  Case-control study | 1) 32 (8 vs 24) pediatric oncology patients. Cases with Pseudomonas infection, matched controls *without* Pseudomonas infection | 1) 7/8 cases reported bath toys use (on which the Pseudomonas strain was identified); 7/24 controls reported bath toys use (on which the Pseudomonas strain was identified). *NB. When ‘controlled for age’ (not reported how’, only a marginally significant association remained with p=0.06.* | 1) Univariate and bivariate analyses, Cornfield method for Odds Ratio and confidence intervals | 1) p=0.004 Odds Ratio 17 (2.2, ∞) | ⨁◯◯◯^A^  VERY LOW |

*A: GRADE: Grade quality assessment bath toy use: design is case-controlled study, inconsistency not serious, indirectness not serious, imprecision serious (downgraded one level because of small study population), publication bias unlikely, downgraded 1 level because of serious risk of bias (selection bias high, attrition bias low, detection bias low, reporting bias unclear, confounding bias high, other bias low).*Buttery, 1998 – Bubble bath use

| **Author, study design** | **No. of participants, total (cases vs controls) & Group definition** | **Results** | **Statistical methods** | **Effect size** | **Quality of evidence** |
| --- | --- | --- | --- | --- | --- |
| 1) Buttery, 1998  Case-control study | 1) 32 (8 vs 24) pediatric oncology patients. Cases with Pseudomonas infection, matched controls *without* Pseudomonas infection | 1) 7/8 cases reported bubble bath use; 9/24 controls reported bubble bath use (Pseudomonas strain not identified). | 1) Univariate and bivariate analyses, Cornfield method for Odds Ratio and confidence intervals | 1B) p=0.014 Odds Ratio 11.7 (1.5, ∞) | ⨁◯◯◯^B^  VERY LOW |

*B: GRADE: Grade quality assessment bubble bath use: design is case-controlled study, inconsistency not serious, indirectness not serious, imprecision serious (downgraded one level because of small study population), publication bias unlikely, downgraded 1 level because of serious risk of bias (selection bias high, attrition bias low, detection bias low, reporting bias unclear, confounding bias high, other bias low).*

Kjellin 2020 – Bath wipes with chlorhexidine

| **Author, study design** | **No. of participants, total (cases vs controls) & Group definition** | **Results** | **Statistical methods** | **Effect size** | **Quality of evidence** |
| --- | --- | --- | --- | --- | --- |
| 1) Kjellin, 2020 RCT | 1) Patients ≤21 years of age who were scheduled to undergo an autologous or allogeneic HCT received either experimental bath wipes (see table 1 for exact composition) or standard bath wipes. | 1A) Experimental bath wipes; colonization of vancomycin-resistant enterococcus in 1/61 patients (2%).  Standard bath wipes; colonization vancomycin-resistant enterococcus in 2/66 patients (3%).   1B) Colonization with multidrug resistant organisms 0 in both groups.  1C) Experimental bath wipes; CLABSI in 0/61 patients (0%).  Standard bath wipes; CLABSI in 2/66 patients (3%). (**Staphylococcus epidermidis & Pseudomonas)* | 1) Fisher’s exact test | 1A) p=1    1B) Not applicable  1C) p=0.50 | ⨁⨁◯◯^C^ LOW |

*C: GRADE: Grade quality assessment bath wipes: design is randomized controlled trial, inconsistency not serious, indirectness not serious, imprecision serious (because of few events and therefore halting the trial), publication bias unlikely, downgraded 1 level because of serious risk of bias (random sequence generation unclear, allocation concealment unclear, performance bias high, detection bias low, attrition bias low, reporting bias unclear, other bias high)*

Raulji, 2015, Zerr, 2020 – Chlorhexidine bathing

| **Author, study design** | **No. of participants, total (cases vs controls) & Group definition** | **Results** | **Statistical methods** | **Effect size** | **Quality of evidence** |
| --- | --- | --- | --- | --- | --- |
| 1) Raulji, 2015  Pre- and post-intervention study | 1) 330 (140 vs 190) pediatric oncology patients.  Daily bathing with chlorhexidine versus no-chlorhexidine bathing | 1A) Total amount of patients getting **any** infection 20/190 children (10,5%) with any infection in pre-intervention group (no chlorhexidine bathing); 12/140 (8.6%) in post-intervention group (chlorhexidine bathing).  1A1) Total amount of **blood stream infections** (BSI) infection per group: 7 incidences in control group, 3 incidences in chlorhexidine group.  1B1) Incidence density (number of occurrences of infection/100 days) in age group 12-21: 3.91 in control group; 0.96 in intervention (chlorhexidine) group.  1B2) Incidence density in age groups 4-7 and 7-12 lower in study group (respectively; 1.49 vs 0.00 and 2.62 vs 1.14).  1B3) In age group 0-4, incidence density of infection **higher** in study group, 2.28 versus 1.31 in control group. | 1A) Not provided    1A1) Not provided   1B1) Not provided   1B2) Not provided      1B3) Not provided | 1A) Not provided  1A1) p=0.70   1B1) p = 0.03   1B2) p-values respectively p=0.07 and p=0.28   1B3) p=0.29 | ⨁◯◯◯^D^  VERY LOW |
| 2) Zerr, 2020  RCT | 2) Pediatric oncology patients with externally tunneled CVCs underwent once-daily bathing for 90 days with either cloths impregnated with mild cleansers or with 2% CHG-impregnated cloths. | 2A) Chlorhexidine group: 5.44 CLABSI per 1000 line days  Control group: 3.1 CLABSI per 1000 line days   2B) Chlorhexidine group: the estimated 90-day cumulative incidence of CLABSI was 34.6% [(95% CI, 25.1-46.4%].  Control group: the estimated 90-day cumulative incidence of CLABSI was 24.1% [95% CI 16.1-35.3%].  2C) Cutaneous adverse events chlorhexidine group: 24% Cutaneous adverse events control group: 15% (When limited to events to be at least possibly related to the intervention, the frequency of events was lower (10% vs 6%). | 2A) Adjusted incidence rate ratio   2B) Log rank test    2C) Not reported | 2A) 1.76 [95% CI 1.00-3.08],  p= 0.049  2B) p=0.091    2C) Not reported | ⨁⨁◯◯^E^ LOW |

*D: GRADE: Grade quality assessment chlorhexidine bathing: design is case-controlled study, inconsistency not serious, indirectness not serious, imprecision not serious, publication bias unlikely, downgraded 2 levels because of very serious risk of bias (selection bias high, attrition bias high, detection bias low, reporting bias unclear, confounding bias high, other bias high).

E: GRADE: Grade quality assessment chlorhexidine bathing: design is randomized controlled trial, inconsistency not serious, indirectness not serious, imprecision not serious, publication bias unlikely, downgraded 2 levels because of very serious risk of bias (random sequence generation low, allocation concealment low, performance bias low, detection bias unclear, attrition bias high, reporting bias unclear, other bias low)*Tramsen 2016 – Restriction in pets

| **Author, study design** | **No. of participants, total (cases vs controls) & Group definition** | **Results** | **Statistical methods** | **Effect size** | **Quality of evidence** |
| --- | --- | --- | --- | --- | --- |
| 1) Tramsen 2016  Retrospective cohort study | 1) 339 pediatric AML patients. Hospitals filled in restriction scores regarding pets and social contacts. | 1A1) When the results were adjusted for gender, age, weight groups, risk stratification and antibiotic prophylaxis, restriction of pets was **not** significantly associated with decreased risk of fever of unknown origin.   1A2) For adjusted results (see above), restriction of pets was **not** significantly associated with decreased risk of bacteremia.   1A3) For adjusted results (see above), restriction of pets was **not** significantly associated with decreased risk of pneumonia.   1A4) For adjusted results (see above), restriction of pets was **not** significantly associated with decreased risk of gastroenteritis. | 1) Poisson regression and associated 95% CI | 1A1) IRR 0.99, 95% CI 0.95-1.03, p=0.59   1A2) IRR 0.99, 95% CI 0.94-1.05, p=0.75  1A3) IRR 0.91, 95% CI 0.82-1.02, p=0.11  1A4) IRR 1.05, 95% CI 0.95-1.17, p=0.316 | ⨁◯◯◯^F^  VERY LOW |

*F: GRADE:* Grade quality assessment *restriction in pets at home:* design is retrospective cohort study, inconsistency not serious, indirectness not serious, imprecision serious, publication bias unlikely, downgraded 1 level because of serious risk of bias (selection bias low, attrition bias low, detection bias low, reporting bias unclear, confounding bias high, other bias low).

Tramsen 2016 – Restriction in social contact (school and kindergarten)

| **Author, study design** | **No. of participants, total (cases vs controls) & Group definition** | **Results** | **Statistical methods** | **Effect *size*** | **Quality of evidence** |
| --- | --- | --- | --- | --- | --- |
| 1) Tramsen 2016  Retrospective cohort study | 1) 339 pediatric AML patients. Hospitals filled in restriction scores regarding pets and social contacts. | 1A1) When the results were adjusted for gender, age, weight groups, risk stratification and antibiotic prophylaxis, restriction of social contact was **not** significantly associated with decreased risk of fever of unknown origin.   1A2) For adjusted results (see above), restriction of social contact was **not** significantly associated with decreased risk of bacteremia.   1A3) For adjusted results (see above), restriction of social contact was **not** significantly associated with decreased risk of pneumonia.   1A4) For adjusted results (see above), restriction of pets was **not** significantly associated with decreased risk of gastroenteritis. | 1) Poisson regression and associated 95% CI | 1A1) IRR 0.99, 95% CI 0.92-1.08, p=0.9    1A2) IRR 1.15, 95% CI 0.99-1.33, p=0.066  1A3) IRR 0.99, 95% CI 0.77-1.28, p=0.96  1A4) IRR 0.94, 95% CI 0.73-1.21, p=0.63 | ⨁◯◯◯^G^  VERY LOW |

*G: GRADE:* Grade quality assessment *restriction in social contacts:* design is retrospective cohort study, inconsistency not serious, indirectness not serious, imprecision serious, publication bias unlikely, downgraded 1 level because of serious risk of bias (selection bias low, attrition bias low, detection bias low, reporting bias unclear, confounding bias high, other bias low).

Robbins 1999 – Swimming

| **Author, study design** | **No. of participants, total (cases vs controls) & Group definition** | **Results** | **Statistical methods** | **Effect *size*** | **Quality of evidence** |
| --- | --- | --- | --- | --- | --- |
| 1) Robbins, 1999  Retrospective cohort study | 1) 95 (49 swimmers vs 46 non-swimmers) pediatric oncology patients with tunneled central venous catheter.  Swimmers (i.e. swimming at least once when catheter was in place) versus non-swimmers | 1A) Total of infections: 34 in swimmers group, 13 in non-swimmer group (Note: baseline imbalances in months of catheter in place).  1B) **Total** infections per month in swimmers group: 34 infections per 843 months of catheter in place (0.04%). In non-swimmers group, 13 infections were reported in 506 months of catheter in place (0.025%).  1B1) **Tunnel or exit** infections per month in the swimmers group: 20 infections per 843 months of catheter in place (0.02%). In non-swimmers group, 8 infections were reported in 506 months of catheter in place (0.016%).  1B2) **Bloodstream infections** per month in the swimmers group: 14 infections per 843 months of catheter in place (0.016%). In non-swimmers group, 5 infections were reported in 506 months of catheter in place (0.009%). | 1A) Not reported    1B) Relative Risk and Chi-squared test    1B1) Relative Risk and Chi-squared test    1B2) Relative Risk and Chi-squared test | 1A) Not reported  1B) RR 1.6, not significant (p-value not reported)  1B1) RR 1.5, not significant (p-value not reported)  1B2) RR 1.7, not significant (p-value not reported) | ⨁◯◯◯^H^  VERY LOW |

*H: GRADE: Grade quality assessment swimming: design is case-controlled study, inconsistency not serious, indirectness not serious, imprecision serious (downgraded one level because of small study population), publication bias unlikely, downgraded 1 level because of serious risk of bias (selection bias unclear, attrition bias high, detection bias low, reporting bias unclear, confounding bias high, other bias high).*

Robbins 1999 – Frequent swimming

| **Author, study design** | **No. of participants, total (cases vs controls) & Group definition** | **Results** | **Statistical methods** | **Effect *size*** | **Quality of evidence** |
| --- | --- | --- | --- | --- | --- |
| 1) Robbins, 1999  Retrospective cohort study | 1) 95 (35 frequent swimmers vs 60 infrequent swimmers) pediatric oncology patients with tunneled central venous catheter.  Frequent swimmers (i.e. at least once a month) versus infrequent or non-swimmers | 1A) 19 catheter-related infections in frequent swimmers; 27 among infrequent/non-swimmers (Note: baseline imbalances in months of catheter in place).  1B) **Total** infections per month in swimmers group: 19 infections per 579 months of catheter in place (0.03%). In infrequent/non-swimmers, 27 infections were reported in 770 months of catheter in place (0.03%).  1B1) **Tunnel or exit** infections per month in the swimmers group: 6 infections per 579 months of catheter in place (0.01%). In infrequent/non-swimmers 16 infections were reported in 770 months of catheter in place (0.02%).  1B2) **Bloodstream infections** per month in the swimmers group: 13 infections per 579 months of catheter in place (0.02%). In infrequent/non-swimmers, 11 infections were reported in 770 months of catheter in place (0.014%). | 1A) Not reported   1) Relative Risk and Chi-squared test   1B1) Relative Risk and Chi-squared test   1B2) Relative Risk and Chi-squared test | 1A) Not reported   1B) RR 0.9, not significant (p-value not reported)  1B1) RR 0.5, not significant (p-value not reported)  1B2) RR 1.6, not significant (p-value not reported) | ⨁◯◯◯^I^  VERY LOW |

*I: GRADE: Grade quality assessment frequent swimming: design is case-controlled study, inconsistency not serious, indirectness not serious, imprecision serious (downgraded one level because of small study population), publication bias unlikely, downgraded 1 level because of serious risk of bias (selection bias unclear, attrition bias high, detection bias low, reporting bias unclear, confounding bias high, other bias high).*

**S7: Evidence-to-decision Frameworks
Evidence to decision framework 1: Bath toy use**

|  | Criteria | Judgements | Research evidence | Additional considerations |
| --- | --- | --- | --- | --- |
| PROBLEM | Is the problem a priority? | ☐ No  ☐ Probably no  ☐ Uncertain  ☐ Probably yes  ■ **Yes** | Improving quality of life has become increasingly important in care for children with cancer. Due to improved survival rates there is an increased focus on morbidity and adverse effects of anti-cancer treatment (1, 2). To prevent adverse health problems, such as infections and bleeding, restrictions in daily life have been defined for children with cancer related to school attendance, travelling with public transport, pets, hygiene measures and swimming (3). However, these social restrictions can severely impair the quality of life of these children (4, 5).  Within the Netherlands, there is large variation in current supportive care practices, including social restrictions (6). The majority of these recommendations regarding social restrictions for children with cancer are not evidence-based. Guidance is necessary in order to provide the best possible care for these children, balancing cautiousness and restrictiveness. |  |
| BENEFITS AND HARMS | What is the overall certainty of this evidence? | ☐ No included studies  ■ **Very low**  ☐ Low  ☐ Moderate  ☐ High | \| Outcome \| Relative importance \| Quality of evidence \| \| \| --- \| --- \| --- \| --- \| \| Number of infections \| Critical \| Very low \|   Summary of findings:  1. Number of infections  1. 7/8 cases reported bath toys use (on which the Pseudomonas strain was identified); 7/24 controls reported bath toys use (on which the Pseudomonas strain was identified). p=0.004, Odds Ratio 17 (2.2, ∞) |  |
|  | Is there important uncertainty about how much people value the main outcomes? | ☐ Important uncertainty or variability  ☐ Possibly important uncertainty or variability  ☐ Probably no important uncertainty or variability  ■ **No important uncertainty or variability**  ☐ No known undesirable outcomes |  | 1. When ‘controlled for age’ (not reported how, only a marginally significant association remained with p=0.06.) |
|  | Are the desirable anticipated effects large? | ☐ No  ■ **Probably no**  ☐ Uncertain  ☐ Probably yes  ☐ Yes  ☐ Varies |  |  |
|  | Are the undesirable anticipated effects small? | ■ **No**  ☐ Probably no  ☐ Uncertain  ☐ Probably yes  ☐ Yes  ☐ Varies |  |  |
|  | Are the desirable effects large relative to undesirable effects? | ☐ No  ■ **Probably no**  ☐Uncertain  ☐ Probably yes  ☐ Yes  ☐ Varies |  |  |
| RESOURCE USE | Are the resources required small? | ☐ No  ☐ Probably no  ☐ Uncertain  ☐ Probably yes  ■ **Yes**  ☐ Varies |  |  |
|  | Is the incremental cost small relative to the net benefits? | ☐ No  ☐ Probably no  ■ **Uncertain**  ☐ Probably yes  ☐ Yes  ☐ Varies |  |  |
| EQUITY | What would be the impact on health inequities? | ☐ Increased  ☐ Probably increased  ■ **Uncertain**  ☐ Probably reduced  ☐ Reduced  ☐ Varies |  | The panel expects that this daily life restriction will not have any effect on health inequities. |
| ACCEPTABILITY | Is the option acceptable to key stakeholders? | ☐ No  ☐ Probably no  ☐ Uncertain  ☐ Probably yes  ■ **Yes**  ☐ Varies |  |  |
| FEASIBILITY | Is the option feasible to implement? | ☐ No  ☐ Probably no  ☐ Uncertain  ☐ Probably yes  ■**Yes**  ☐ Varies |  |  |

**Conclusion EtD:**

| PROBLEM |  |
| --- | --- |
| Is the problem a priority? | Yes |
| BENEFITS AND HARMS |  |
| What is the overall certainty of this evidence? | Very low quality |
| Is there important uncertainty about how much people value the main outcomes? | No important uncertainty or variability |
| Are the desirable anticipated effects large? | Probably no |
| Are the undesirable anticipated effects small? | No |
| Are the desirable effects large relative to undesirable effects? | Probably no |
| RESOURCE USE |  |
| Are the resources required small? | Yes |
| Is the incremental cost small relative to the net benefits? | Uncertain |
| EQUITY |  |
| What would be the impact on health inequities? | Uncertain |
| ACCEPTABILITY |  |
| Is the option acceptable to key stakeholders? | Yes |
| FEASABILITY |  |
| Is the option feasible to implement? | Yes |

**Overall conclusions:**

| Balance of consequences | | | | |
| --- | --- | --- | --- | --- |
| **Undesirable consequences  *clearly outweigh*  desirable consequences in most settings**  ■ | Undesirable consequences *probably outweigh*  desirable consequences in most settings  ☐ | The balance between  desirable and undesirable consequences  *is closely balanced or uncertain*  ☐ | Desirable consequences  *probably outweigh* undesirable consequences in most settings  ☐ | Desirable consequences  *clearly outweigh*  undesirable consequences in most settings  ☐ |

**Recommendation:**

| Type of recommendation – Bath toy use | | | |
| --- | --- | --- | --- |
| **We recommend against**  **offering this option**  ■ | We suggest not offering  this option  ☐ | We suggest offering  this option  ☐ | We recommend offering  this option  ☐ |
| Recommendation (text) | **We recommend against the use of bath toys that have a reservoir (in which water can be retained) or bath toys that cannot be dried thoroughly. (STRONG recommendation, VERY LOW quality of evidence**) | | |
| Justification | One study in pediatric oncology patients was found. In this study (7), significantly more bath toy use was reported in the group infected with *Pseudomonas* compared to the group without *Pseudomonas* infection (note, significance was lost when correcting for age, also, Pseudomonas strain was identified in the culture of bath toys). This study is small and retrospective, but it does show a (possible) effect of the use of bath toys, which is supported by our expert opinions.  The guideline panel agrees that bath toys with a reservoir in which water can be retained should not be used in children with cancer. The still standing water in the reservoir, for example in the inside of a bath toy as in the included study, is a reservoir for several bacteria like *Pseudomonas*, which can cause severe infections in these children. Also, toys that cannot be dried thoroughly are prone to formation of for bacteria and should therefore not be used. | | |
| Subgroup considerations | Not applicable. | | |
| Implementation considerations | It is not necessary to dispose all bath toys for (younger) children with cancer during their treatment. The panel agrees that if toys can be dried thoroughly and if there is no reservoir in which water can be retained, the toys are probably not an infectious risk and can be used safely.  Note that this also accounts for sponges, towels and other items that become wet during showering or bathing. As long as water is not retained in these items, we believe the infectious risk remains low. | | |
| Monitoring and evaluation | Not applicable. | | |
| Research priorities | Not applicable. | | |

**Evidence to decision framework 2: Bubble bath use**

|  | Criteria | Judgements | Research evidence | Additional considerations |
| --- | --- | --- | --- | --- |
| PROBLEM | Is the problem a priority? | ☐ No  ☐ Probably no  ☐ Uncertain  ☐ Probably yes  ■ **Yes** | Improving quality of life has become increasingly important in care for children with cancer. Due to improved survival rates there is an increased focus on morbidity and adverse effects of anti-cancer treatment (1, 2). To prevent adverse health problems, such as infections and bleeding, restrictions in daily life have been defined for children with cancer related to school attendance, travelling with public transport, pets, hygiene measures and swimming (3). However, these social restrictions can severely impair the quality of life of these children (4, 5).  Within the Netherlands, there is large variation in current supportive care practices, including social restrictions (6). The majority of these recommendations regarding social restrictions for children with cancer are not evidence-based. Guidance is necessary in order to provide the best possible care for these children, balancing cautiousness and restrictiveness. |  |
| BENEFITS AND HARMS | What is the overall certainty of this evidence? | ☐ No included studies  ■ **Very low**  ☐ Low  ☐ Moderate  ☐ High | \| Outcome \| Relative importance \| Quality of evidence \| \| \| --- \| --- \| --- \| --- \| \| Number of infections \| Critical \| Very low \|   Summary of findings:  1. Number of infections  1. 7/8 cases reported bubble bath use; 9/24 controls reported bubble bath use (Pseudomonas strain not identified). p=0.014, Odds Ratio 11.7 (1.5, ∞) |  |
|  | Is there important uncertainty about how much people value the main outcomes? | ☐ Important uncertainty or variability  ☐ Possibly important uncertainty or variability  ☐ Probably no important uncertainty or variability  ■ **No important uncertainty or variability**  ☐ No known undesirable outcomes |  |  |
|  | Are the desirable anticipated effects large? | ☐ No  ☐ Probably no  ☐ Uncertain  ■ **Probably yes**  ☐ Yes  ☐ Varies |  |  |
|  | Are the undesirable anticipated effects small? | ■ **No**  ☐ Probably no  ☐ Uncertain  ☐ Probably yes  ☐ Yes  ☐ Varies |  |  |
|  | Are the desirable effects large relative to undesirable effects? | ☐ No  ☐ Probably no  ■**Uncertain**  ☐ Probably yes  ☐ Yes  ☐ Varies |  | Different for bubble bath use in public accessible baths. There, undesirable effects > desirable effects, for bubble bath use at home desirable effects > undesirable effects. |
| RESOURCE USE | Are the resources required small? | ☐ No  ☐ Probably no  ☐ Uncertain  ☐ Probably yes  ■ **Yes**  ☐ Varies |  |  |
|  | Is the incremental cost small relative to the net benefits? | ☐ No  ☐ Probably no  ■ **Uncertain**  ☐ Probably yes  ☐ Yes  ☐ Varies |  |  |
| EQUITY | What would be the impact on health inequities? | ☐ Increased  ☐ Probably increased  ■ **Uncertain**  ☐ Probably reduced  ☐ Reduced  ☐ Varies |  | The panel expects that this daily life restriction will not have any effect on health inequities. |
| ACCEPTABILITY | Is the option acceptable to key stakeholders? | ☐ No  ☐ Probably no  ☐ Uncertain  ☐ Probably yes  ■ **Yes**  ☐ Varies |  |  |
| FEASIBILITY | Is the option feasible to implement? | ☐ No  ☐ Probably no  ☐ Uncertain  ☐ Probably yes  ■**Yes**  ☐ Varies |  |  |

**Conclusion EtD:**

| PROBLEM |  |
| --- | --- |
| Is the problem a priority? | Yes |
| BENEFITS AND HARMS |  |
| What is the overall certainty of this evidence? | Very low quality |
| Is there important uncertainty about how much people value the main outcomes? | No important uncertainty or variability |
| Are the desirable anticipated effects large? | Probably yes |
| Are the undesirable anticipated effects small? | No |
| Are the desirable effects large relative to undesirable effects? | Uncertain |
| RESOURCE USE |  |
| Are the resources required small? | Yes |
| Is the incremental cost small relative to the net benefits? | Uncertain |
| EQUITY |  |
| What would be the impact on health inequities? | Uncertain |
| ACCEPTABILITY |  |
| Is the option acceptable to key stakeholders? | Yes |
| FEASABILITY |  |
| Is the option feasible to implement? | Yes |

**Overall conclusions:**

| Balance of consequences | | | | |
| --- | --- | --- | --- | --- |
| Undesirable consequences  *clearly outweigh*  desirable consequences in most settings  ☐ | **Undesirable consequences *probably outweigh*  desirable consequences in most settings**  ■ | The balance between  desirable and undesirable consequences  *is closely balanced or uncertain*  ☐ | Desirable consequences  *probably outweigh* undesirable consequences in most settings  ☐ | Desirable consequences  *clearly outweigh*  undesirable consequences in most settings  ☐ |

**Recommendation:**

| Type of recommendation – Bubble bath use | | | |
| --- | --- | --- | --- |
| We recommend against  offering this option  ☐ | **We suggest not offering**  **this option**  ■ | We suggest offering  this option  ☐ | We recommend offering  this option  ☐ |
| Recommendation (text) | **We suggest not to use warm publically accessible bubble baths. (WEAK recommendation, VERY LOW quality of evidence)**  **We believe the use of a bubble bath at home is allowed, as long as the bath can be cleaned thoroughly and water is refreshed completely after every bath. (WEAK recommendation, EXPERT evidence)** | | |
| Justification | One study in pediatric oncology patients was found. In this study (7), significantly more bubble bath use was reported in the group infected with *Pseudomonas* compared to the group without *Pseudomonas* infection (note, the *Pseudomonas* strain was not identified in culture of bubble bath water). This study is small and retrospective, but it does show a (possible) effect of the use of bubble baths on *Pseudomonas* infection.  Firstly, the guideline panel suggests not to use warm bubble baths in for example public swimming pools, saunas or other accommodations. We believe the infectious risk in these types of bubble baths is high because of the amount of people that enter the bubble baths, the constant high temperature of the bubble baths that form a good environment for bacteria and most importantly the fact that, for these publically accessible bubble baths, water is not frequently refreshed. Therefore we suggest not to use these types of -publically accessible warm bubble baths. | | |
| Subgroup considerations | The guideline panel agrees that there are also possible harms for the use of bubble baths at home or at for example a vacation accommodation. However, these baths, unlike the publically accessible bubble baths, are not visited by other people and water can be refreshed easily. Therefore, the guideline panel believes that if the bath can be cleaned properly before the use of the bath, and water can be completely refreshed, the use of a bubble bath at home (or at a vacation accommodation) is allowed. | | |
| Implementation considerations | Not applicable. | | |
| Monitoring and evaluation | Not applicable. | | |
| Research priorities | Not applicable. | | |

**Evidence to decision framework 3: Chlorhexidine use**

|  | Criteria | Judgements | Research evidence | Additional considerations |
| --- | --- | --- | --- | --- |
| PROBLEM | Is the problem a priority? | ☐ No  ☐ Probably no  ☐ Uncertain  ☐ Probably yes  ■ **Yes** | Improving quality of life has become increasingly important in care for children with cancer. Due to improved survival rates there is an increased focus on morbidity and adverse effects of anti-cancer treatment (1, 2). To prevent adverse health problems, such as infections and bleeding, restrictions in daily life have been defined for children with cancer related to school attendance, travelling with public transport, pets, hygiene measures and swimming (3). However, these social restrictions can severely impair the quality of life of these children (4, 5).  Within the Netherlands, there is large variation in current supportive care practices, including social restrictions (6). The majority of these recommendations regarding social restrictions for children with cancer are not evidence-based. Guidance is necessary in order to provide the best possible care for these children, balancing cautiousness and restrictiveness. |  |
| BENEFITS AND HARMS | What is the overall certainty of this evidence? | ☐ No included studies  ■ **Very low**  ☐ Low  ☐ Moderate  ☐ High | \| Outcome \| Relative importance \| Quality of evidence \| \| \| --- \| --- \| --- \| --- \| \| Number of infections (Bath wipes) \| Critical \| Low \| \| Number of infections (Chlorhexidine bathing) \| Critical \| Very low \| \| Adverse events \| Important \| Very low \|   Summary of findings:  1. Number of infections (Chlorhexidine bath wipes) 1A. Experimental bath wipes; colonization of vancomycin-resistant enterococcus in 1/61 patients (2%).  Standard bath wipes; colonization vancomycin-resistant enterococcus in 2/66 patients (3%). p=1.  1B. Colonization with multidrug resistant organisms 0 in both groups.  1C. Experimental bath wipes; CLABSI in 0/61 patients (0%). Standard bath wipes; CLABSI in 2/66 patients (3%). (**Staphylococcus epidermidis & Pseudomonas).* p=0.50  2. Number of infections (Chlorhexidine bathing) 2.1A. Total amount of patients getting **any** infection 20/190 children (10,5%) with any infection in pre-intervention group (no chlorhexidine bathing); 12/140 (8.6%) in post-intervention group (chlorhexidine bathing). Effect size not provided.  2.1A. Total amount of **blood stream infections** (BSI) infection per group: 7 incidences in control group, 3 incidences in chlorhexidine group. p=0.70.  2.1B1. Incidence density (number of occurrences of infection/100 days) in age group 12-21: 3.91 in control group; 0.96 in intervention (chlorhexidine) group. p=0.03.  2.1B2. Incidence density in age groups 4-7 and 7-12 lower in study group (respectively; 1.49 vs 0.00 and 2.62 vs 1.14). p-values respectively p=0.07 and p=0.28. 2.1B3. In age group 0-4, incidence density of infection **higher** in study group, 2.28 versus 1.31 in control group. p=0.29.  2.2A. Chlorhexidine group: 5.44 CLABSI per 1000 line days, control group: 3.1 CLABSI per 1000 line days 1.76 [95% CI 1.00-3.08]. p= 0.049 2.2B. Chlorhexidine group: the estimated 90-day cumulative incidence of CLABSI was 34.6% [(95% CI, 25.1-46.4%]. Control group: the estimated 90-day cumulative incidence of CLABSI was 24.1% [95% CI 16.1-35.3%]. p=0.091.   3. Adverse events (Chlorhexidine bathing)  3. Cutaneous adverse events chlorhexidine group: 24%. Cutaneous adverse events control group: 15% (When limited to events to be at least possibly related to the intervention, the frequency of events was lower (10% vs 6%). Effect size not provided. |  |
|  | Is there important uncertainty about how much people value the main outcomes? | ☐ Important uncertainty or variability  ☐ Possibly important uncertainty or variability  ☐ Probably no important uncertainty or variability  ■ **No important uncertainty or variability**  ☐ No known undesirable outcomes |  |  |
|  | Are the desirable anticipated effects large? | ☐ No  ■ **Probably no**  ☐ Uncertain  ☐ Probably yes  ☐ Yes  ☐ Varies |  |  |
|  | Are the undesirable anticipated effects small? | ☐ No  ■ **Probably no**  ☐ Uncertain  ☐ Probably yes  ☐ Yes  ☐ Varies |  |  |
|  | Are the desirable effects large relative to undesirable effects? | ☐ No  ■ **Probably no**  ☐ Uncertain  ☐ Probably yes  ☐ Yes  ☐ Varies |  |  |
| RESOURCE USE | Are the resources required small? | ☐ No  ■ **Probably no**  ☐ Uncertain  ☐ Probably yes  ☐ Yes  ☐ Varies |  |  |
|  | Is the incremental cost small relative to the net benefits? | ☐ No  ■**Probably no**  ☐ Uncertain  ☐ Probably yes  ☐ Yes  ☐ Varies |  |  |
| EQUITY | What would be the impact on health inequities? | ☐ Increased  ■**Probably increased**  ☐ Uncertain  ☐ Probably reduced  ☐ Reduced  ☐ Varies |  | If this intervention is not (completely) reimbursed by health care insurance, there could be an increased impact on health inequities. |
| ACCEPTABILITY | Is the option acceptable to key stakeholders? | ☐ No  ☐ Probably no  ☐ Uncertain  ■ **Probably yes**  ☐ Yes  ☐ Varies |  |  |
| FEASIBILITY | Is the option feasible to implement? | ☐ No  ☐ Probably no  ☐ Uncertain  ■ **Probably yes**  ☐ Yes  ☐ Varies |  |  |

**Conclusion EtD:**

| PROBLEM |  |
| --- | --- |
| Is the problem a priority? | Yes |
| BENEFITS AND HARMS |  |
| What is the overall certainty of this evidence? | Very low quality |
| Is there important uncertainty about how much people value the main outcomes? | No important uncertainty or variability |
| Are the desirable anticipated effects large? | Probably no |
| Are the undesirable anticipated effects small? | Probably no |
| Are the desirable effects large relative to undesirable effects? | Probably no |
| RESOURCE USE |  |
| Are the resources required small? | Probably no |
| Is the incremental cost small relative to the net benefits? | Probably no |
| EQUITY |  |
| What would be the impact on health inequities? | Probably increased |
| ACCEPTABILITY |  |
| Is the option acceptable to key stakeholders? | Probably yes |
| FEASABILITY |  |
| Is the option feasible to implement? | Probably yes |

**Overall conclusions:**

| Balance of consequences | | | | |
| --- | --- | --- | --- | --- |
| Undesirable consequences  *clearly outweigh*  desirable consequences in most settings  ☐ | **Undesirable consequences *probably outweigh*  desirable consequences in most settings**  ■ | The balance between  desirable and undesirable consequences  *is closely balanced or uncertain*  ☐ | Desirable consequences  *probably outweigh* undesirable consequences in most settings  ☐ | Desirable consequences  *clearly outweigh*  undesirable consequences in most settings  ☐ |

**Recommendation:**

| Type of recommendation – Chlorhexidine use | | | |
| --- | --- | --- | --- |
| We recommend against  offering this option  ☐ | **We suggest not offering**  **this option**  ■ | We suggest offering  this option  ☐ | We recommend offering  this option  ☐ |
| Recommendation (text) | **We suggest not to use chlorhexidine bathing or other bath wipes as it does not seem to have an added value to basic hygiene measures. (WEAK recommendation, VERY LOW quality of evidence)** | | |
| Justification | *1.1 Bath wipes* One study (10) in pediatric oncology patients was found. In conclusion, no significant differences in prevalence of infections were seen in the experimental bath wipes group versus the standard bath wipes group. The guideline panel does not see any reason to suggest the use of these types of bath wipes, as the panel does not see any added value to basic hygiene measures.  This recommendation is based on evidence on bath wipes with ingredients such as allantoin, colloidal silver, preservatives, vitamin E, aloe, and lauryl glucoside (10). There was no evidence for any other types of bath wipes, and therefore we feel that bath wipes *in general* would not have any added value. Therefore, the panel suggests not to use bath wipes as it does not seem to have an added value to basic hygiene measures.  *1.2 Chlorhexidine bathing* Two studies in pediatric oncology patients were found. In one study (8), overall, no significant differences in prevalence of infections between patients with versus without chlorhexidine bathing were found. In this study, significantly lower prevalence of infections in patients with versus without chlorhexidine bathing in specific age group 12-21 years was reported. We believe that this specific reported lower prevalence of infections in the specific age group 12-21 years is mostly coincidental. The age groups were not predefined in the study and therefore it is difficult to assess the validity this outcome. Possibly, this is an age group in which basic hygiene measures might not be followed strictly, and that this result is possibly the result of a different intervention (namely ‘regular basic hygiene measures’ rather than ‘chlorhexidine bathing’). Also in all the other age groups, no differences were seen between the two groups.  In one study (9), significant differences in prevalence of infections were seen in the chlorhexidine bathing group versus the control group were reported. This study, however, showed higher CLABSI rates in the chlorhexidine group. This study was of low quality and was stopped early because of poor accrual but does not support the use of routine chlorhexidine bathing in children with cancer.  Summarizing, two studies (8, 9) show inconsistent results as one of the studies even pointed towards more CLABSI in the chlorhexidine group, and the outcome from the other study that does show a significant difference in favor of the chlorhexidine group, is difficult to interpret. The guideline panel does not see any added value for chlorhexidine bathing, and we consider it more of a burden to these children. Therefore, the panel suggests not to use chlorhexidine bathing as it does not seem to have an added value to basic hygiene measures.  In summary, the guideline panel feels that both chlorhexidine bathing and bath wipes do not have an added value and that just following basic hygiene measures should be sufficient. | | |
| Subgroup considerations | Not applicable. | | |
| Implementation considerations | Not applicable. | | |
| Monitoring and evaluation | Not applicable. | | |
| Research priorities | Not applicable. | | |

**Evidence to decision framework 4: Environmental factors (including sandbox)**

|  | Criteria | Judgements | Research evidence | Additional considerations |
| --- | --- | --- | --- | --- |
| PROBLEM | Is the problem a priority? | ☐ No  ☐ Probably no  ☐ Uncertain  ☐ Probably yes  ■ **Yes** | Improving quality of life has become increasingly important in care for children with cancer. Due to improved survival rates there is an increased focus on morbidity and adverse effects of anti-cancer treatment (1, 2). To prevent adverse health problems, such as infections and bleeding, restrictions in daily life have been defined for children with cancer related to school attendance, travelling with public transport, pets, hygiene measures and swimming (3). However, these social restrictions can severely impair the quality of life of these children (4, 5).  Within the Netherlands, there is large variation in current supportive care practices, including social restrictions (6). The majority of these recommendations regarding social restrictions for children with cancer are not evidence-based. Guidance is necessary in order to provide the best possible care for these children, balancing cautiousness and restrictiveness. |  |
| BENEFITS AND HARMS | What is the overall certainty of this evidence? | ■ **No included studies**  ☐ Very low  ☐ Low  ☐ Moderate  ☐ High | No evidence in pediatric oncology patients was identified. However, a recommendation by the ASCO and IDSA (20) guideline was used for the decision by the guideline panel. The guideline panel strongly agrees with the recommendation from the ASCO and IDSA guideline (20), as we agree that the stated environmental sites (20), indeed could contain high levels of fungal spores and could therefore be a potential danger. Although this recommendation was not specifically made for children, we believe that it is also applicable to them. | The guideline panel specifically made a recommendation about playing in the sandbox, as this is a clinically relevant subject for parents and children. No evidence in pediatric oncology patients or other guidelines were identified. The guideline panel believes that children with cancer should be allowed to play in the sandbox, either at home, at the playground or at school, as long as they carefully consider their hand hygiene. |
|  | Is there important uncertainty about how much people value the main outcomes? | ☐ Important uncertainty or variability  ☐ Possibly important uncertainty or variability  ■ **Probably no important uncertainty or variability**  ☐ No important uncertainty or variability  ☐ No known undesirable outcomes |  |  |
|  | Are the desirable anticipated effects large? | ☐ No  ☐ Probably no  ☐ Uncertain  ☐ Probably yes  ☐ Yes  ■ **Varies** |  | Depends on the topic, playing in the sandbox can increase quality of life, but this is less important regarding exposure to soil through gardening. |
|  | Are the undesirable anticipated effects small? | ☐ No  ■ **Probably no**  ☐ Uncertain  ☐ Probably yes  ☐ Yes  ☐ Varies |  |  |
|  | Are the desirable effects large relative to undesirable effects? | ☐ No  ☐ Probably no  ☐Uncertain  ☐ Probably yes  ☐ Yes  ■ **Varies** |  |  |
| RESOURCE USE | Are the resources required small? | ☐ No  ☐ Probably no  ☐ Uncertain  ■**Probably yes**  ☐ Yes  ☐ Varies |  |  |
|  | Is the incremental cost small relative to the net benefits? | ☐ No  ☐ Probably no  ☐ Uncertain  ☐ Probably yes  ☐ Yes  ■ **Varies** |  |  |
| EQUITY | What would be the impact on health inequities? | ☐ Increased  ☐ Probably increased  ■ **Uncertain**  ☐ Probably reduced  ☐ Reduced  ☐ Varies |  | The panel expects that this daily life restriction will not have any effect on health inequities.   The only scenario possible is when children become sick when their parents are in a complete construction site of their house. This is such a specific situation that no specific recommendations are made. |
| ACCEPTABILITY | Is the option acceptable to key stakeholders? | ☐ No  ☐ Probably no  ☐ Uncertain  ■**Probably yes**  ☐ Yes  ☐ Varies |  |  |
| FEASIBILITY | Is the option feasible to implement? | ☐ No  ☐ Probably no  ☐ Uncertain  ■ **Probably yes**  ☐Yes  ☐ Varies |  |  |

**Conclusion EtD:**

| PROBLEM |  |
| --- | --- |
| Is the problem a priority? | Yes |
| BENEFITS AND HARMS |  |
| What is the overall certainty of this evidence? | No included studies |
| Is there important uncertainty about how much people value the main outcomes? | No important uncertainty or variability |
| Are the desirable anticipated effects large? | Varies |
| Are the undesirable anticipated effects small? | Probably no |
| Are the desirable effects large relative to undesirable effects? | Varies |
| RESOURCE USE |  |
| Are the resources required small? | Probably yes |
| Is the incremental cost small relative to the net benefits? | Varies |
| EQUITY |  |
| What would be the impact on health inequities? | Uncertain |
| ACCEPTABILITY |  |
| Is the option acceptable to key stakeholders? | Probably yes |
| FEASABILITY |  |
| Is the option feasible to implement? | Probably yes |

**Overall conclusions:**

| Balance of consequences | | | | |
| --- | --- | --- | --- | --- |
| Undesirable consequences  *clearly outweigh*  desirable consequences in most settings  ☐ | Undesirable consequences *probably outweigh*  desirable consequences in most settings  ☐ | The balance between  desirable and undesirable consequences  *is closely balanced or uncertain*  ☐ | **Desirable consequences  *probably outweigh* undesirable consequences in most settings**  ■ | Desirable consequences  *clearly outweigh*  undesirable consequences in most settings  ☐ |

**Recommendation:**

| Type of recommendation – Environmental factors (including sandbox) | | | |
| --- | --- | --- | --- |
| We strongly believe not to  ☐ | We believe not to  ☐ | **We believe that**  ■ | We strongly believe that  ☐ |
| Recommendation (text) | **We recommend that children with cancer and neutropenia should avoid prolonged contact with environments that have high concentrations of fungal spores (i.e. construction or demolition sites, exposure to soil through gardening or digging, household renovation). (STRONG recommendation, ASCO guideline) (1)**  **We believe that children with cancer can play in the sandbox as long as they consider their regular hand hygiene. (WEAK recommendation, EXPERT evidence)** | | |
| Justification | No evidence in pediatric oncology patients was identified. However, a recommendation by the ASCO and IDSA (20) guideline was used for the decision by the guideline panel. The guideline panel strongly agrees with the recommendation from the ASCO and IDSA guideline (20), as we agree that the stated environmental sites (20), indeed could contain high levels of fungal spores and could therefore be a potential danger. Although this recommendation was not specifically made for children, we believe that it is also applicable to them.  The guideline panel specifically made a recommendation about playing in the sandbox, as this is a relevant subject for parents and children. No evidence in pediatric oncology patients or other guidelines were identified. The guideline panel believes that children with cancer should be allowed to play in the sandbox, either at home, at the playground or at school, as long as they carefully consider their hand hygiene. | | |
| Subgroup considerations | Not applicable. | | |
| Implementation considerations | Not applicable. | | |
| Monitoring and evaluation | Not applicable. | | |
| Research priorities | Not applicable. | | |

**Evidence to decision framework 5: Flowers**

|  | Criteria | Judgements | Research evidence | Additional considerations |
| --- | --- | --- | --- | --- |
| PROBLEM | Is the problem a priority? | ☐ No  ☐ Probably no  ☐ Uncertain  ☐ Probably yes  ■ **Yes** | Improving quality of life has become increasingly important in care for children with cancer. Due to improved survival rates there is an increased focus on morbidity and adverse effects of anti-cancer treatment (1, 2). To prevent adverse health problems, such as infections and bleeding, restrictions in daily life have been defined for children with cancer related to school attendance, travelling with public transport, pets, hygiene measures and swimming (3). However, these social restrictions can severely impair the quality of life of these children (4, 5).  Within the Netherlands, there is large variation in current supportive care practices, including social restrictions (6). The majority of these recommendations regarding social restrictions for children with cancer are not evidence-based. Guidance is necessary in order to provide the best possible care for these children, balancing cautiousness and restrictiveness. |  |
| BENEFITS AND HARMS | What is the overall certainty of this evidence? | ■ **No included studies**  ☐ Very low  ☐ Low  ☐ Moderate  ☐ High | No evidence in pediatric oncology patients was found.   Therefore, the recommendation is based on expert opinions. The guideline panel believes that indoor flowers and plants at home should be allowed. We believe the risk of infection of just having plants or flowers in the house, is very minimal. The panel does suggest additional hygiene measures, such as refreshing the water of the flowers regularly, and proposes that the children do not play with or help cleaning the soil of the plants.  Again, basic hygiene measures should be appropriate to minimize the risk of infections through plants and flowers. Therefore, we believe that indoor flowers or plants at home should be allowed. | We believe that this also accounts for a Christmas tree and we see no reason why this should not be allowed in the house. |
|  | Is there important uncertainty about how much people value the main outcomes? | ☐ Important uncertainty or variability  ☐ Possibly important uncertainty or variability  ☐ Probably no important uncertainty or variability  ■ **No important uncertainty or variability**  ☐ No known undesirable outcomes |  |  |
|  | Are the desirable anticipated effects large? | ☐ No  ☐ Probably no  ☐ Uncertain  ■ **Probably yes**  ☐ Yes  ☐ Varies |  |  |
|  | Are the undesirable anticipated effects small? | ☐ No  ☐ Probably no  ☐ Uncertain  ☐ Probably yes  ■ **Yes**  ☐ Varies |  |  |
|  | Are the desirable effects large relative to undesirable effects? | ☐ No  ☐ Probably no  ☐Uncertain  ■ **Probably yes**  ☐ Yes  ☐ Varies |  |  |
| RESOURCE USE | Are the resources required small? | ☐ No  ☐ Probably no  ☐ Uncertain  ☐ Probably yes  ■ **Yes**  ☐ Varies |  |  |
|  | Is the incremental cost small relative to the net benefits? | ☐ No  ☐ Probably no  ☐ Uncertain  ☐ Probably yes  ■ **Yes**  ☐ Varies |  |  |
| EQUITY | What would be the impact on health inequities? | ☐ Increased  ☐ Probably increased  **■ Uncertain**  ☐ Probably reduced  ☐ Reduced  ☐ Varies |  | The panel expects that this daily life restriction will not have any effect on health inequities. |
| ACCEPTABILITY | Is the option acceptable to key stakeholders? | ☐ No  ☐ Probably no  ☐ Uncertain  ☐ Probably yes  ■ **Yes**  ☐ Varies |  |  |
| FEASIBILITY | Is the option feasible to implement? | ☐ No  ☐ Probably no  ☐ Uncertain  ☐ Probably yes  ■**Yes**  ☐ Varies |  |  |

**Conclusion EtD:**

| PROBLEM |  |
| --- | --- |
| Is the problem a priority? | Yes |
| BENEFITS AND HARMS |  |
| What is the overall certainty of this evidence? | No included studies |
| Is there important uncertainty about how much people value the main outcomes? | No important uncertainty or variability |
| Are the desirable anticipated effects large? | Probably yes |
| Are the undesirable anticipated effects small? | Yes |
| Are the desirable effects large relative to undesirable effects? | Probably yes |
| RESOURCE USE |  |
| Are the resources required small? | Yes |
| Is the incremental cost small relative to the net benefits? | Yes |
| EQUITY |  |
| What would be the impact on health inequities? | Uncertain |
| ACCEPTABILITY |  |
| Is the option acceptable to key stakeholders? | Yes |
| FEASABILITY |  |
| Is the option feasible to implement? | Yes |

**Overall conclusions:**

| Balance of consequences | | | | |
| --- | --- | --- | --- | --- |
| Undesirable consequences  *clearly outweigh*  desirable consequences in most settings  ☐ | Undesirable consequences *probably outweigh*  desirable consequences in most settings  ☐ | The balance between  desirable and undesirable consequences  *is closely balanced or uncertain*  ☐ | Desirable consequences  *probably outweigh* undesirable consequences in most settings  ☐ | **Desirable consequences  *clearly outweigh*  undesirable consequences in most settings**  ■ |

**Recommendation:**

| Type of recommendation – Flowers | | | |
| --- | --- | --- | --- |
| We strongly believe not to  ☐ | We believe not to  ☐ | We believe that  ☐ | **We strongly believe that**  ■ |
| Recommendation (text) | **We strongly believe that indoor flowers or plants at home should be allowed. (STRONG recommendation, EXPERT evidence)** | | |
| Justification | No evidence in pediatric oncology patients was found. Therefore, the recommendation is based on expert opinions. The guideline panel believes that indoor flowers and plants at home should be allowed. We believe the risk of infection of just having plants or flowers in the house, is very minimal. | | |
| Subgroup considerations | The panel does suggest additional hygiene measures, such as refreshing the water of the flowers often, and proposes that the children do not play with or help cleaning the soil of the plants. We believe that this also accounts for a Christmas tree and we see no reason why this should not be allowed in the house. | | |
| Implementation considerations | Basic hygiene measures should be appropriate to minimize the risk of infections through plants and flowers. Therefore, we believe that indoor flowers or plants at home should be allowed. | | |
| Monitoring and evaluation | Not applicable. | | |
| Research priorities | Not applicable. | | |

**Evidence to decision framework 6: Events with altitude or pressure differences**

|  | Criteria | Judgements | Research evidence | Additional considerations |
| --- | --- | --- | --- | --- |
| PROBLEM | Is the problem a priority? | ☐ No  ☐ Probably no  ☐ Uncertain  ☐ Probably yes  ■ **Yes** | Improving quality of life has become increasingly important in care for children with cancer. Due to improved survival rates there is an increased focus on morbidity and adverse effects of anti-cancer treatment (1, 2). To prevent adverse health problems, such as infections and bleeding, restrictions in daily life have been defined for children with cancer related to school attendance, travelling with public transport, pets, hygiene measures and swimming (3). However, these social restrictions can severely impair the quality of life of these children (4, 5).  Within the Netherlands, there is large variation in current supportive care practices, including social restrictions (6). The majority of these recommendations regarding social restrictions for children with cancer are not evidence-based. Guidance is necessary in order to provide the best possible care for these children, balancing cautiousness and restrictiveness. |  |
| BENEFITS AND HARMS | What is the overall certainty of this evidence? | ■ **No included studies**  ☐ Very low  ☐ Low  ☐ Moderate  ☐ High | No evidence in pediatric oncology patients was found. Therefore, the recommendation is based on expert opinions.  The guideline panel believes that children with cancer can perform high or low altitude events such as going on a plane or scuba diving. We believe that children in a stable phase of their treatment without severe neutropenia or thrombocytopenia, should be allowed to perform this events. We see no obvious reasons why these events with altitude or pressure differences should be contraindicated in stable children.  However, this should always be a careful consideration for the child as an individual, and therefore this always needs to be discussed and be allowed by the treating physician. |  |
|  | Is there important uncertainty about how much people value the main outcomes? | ☐ Important uncertainty or variability  ☐ Possibly important uncertainty or variability  ■ **Probably no important uncertainty or variability**  ☐ No important uncertainty or variability  ☐ No known undesirable outcomes |  |  |
|  | Are the desirable anticipated effects large? | ☐ No  ☐ Probably no  ☐ Uncertain  ■ **Probably yes**  ☐ Yes  ☐ Varies |  |  |
|  | Are the undesirable anticipated effects small? | ☐ No  ☐ Probably no  ☐ Uncertain  ■ **Probably yes**  ☐ Yes  ☐ Varies |  |  |
|  | Are the desirable effects large relative to undesirable effects? | ☐ No  ☐ Probably no  ☐Uncertain  ■ **Probably yes**  ☐ Yes  ☐ Varies |  |  |
| RESOURCE USE | Are the resources required small? | ☐ No  ☐ Probably no  ☐ Uncertain  ☐ Probably yes  ■ **Yes**  ☐ Varies |  |  |
|  | Is the incremental cost small relative to the net benefits? | ☐ No  ☐ Probably no  ☐ Uncertain  ■ **Probably yes**  ☐ Yes  ☐ Varies |  |  |
| EQUITY | What would be the impact on health inequities? | ☐ Increased  ☐ Probably increased  ■ **Uncertain**  ☐ Probably reduced  ☐ Reduced  ☐ Varies |  | The panel expects that this daily life restriction will not have any effect on health inequities. |
| ACCEPTABILITY | Is the option acceptable to key stakeholders? | ☐ No  ☐ Probably no  ☐ Uncertain  ■ **Probably yes**  ☐ Yes  ☐ Varies |  |  |
| FEASIBILITY | Is the option feasible to implement? | ☐ No  ☐ Probably no  ☐ Uncertain  ■ **Probably yes**  ☐Yes  ☐ Varies |  |  |

**Conclusion EtD:**

| PROBLEM |  |
| --- | --- |
| Is the problem a priority? | Yes |
| BENEFITS AND HARMS |  |
| What is the overall certainty of this evidence? | No included studies |
| Is there important uncertainty about how much people value the main outcomes? | No important uncertainty or variability |
| Are the desirable anticipated effects large? | Probably yes |
| Are the undesirable anticipated effects small? | Probably yes |
| Are the desirable effects large relative to undesirable effects? | Probably yes |
| RESOURCE USE |  |
| Are the resources required small? | Yes |
| Is the incremental cost small relative to the net benefits? | Probably yes |
| EQUITY |  |
| What would be the impact on health inequities? | Uncertain |
| ACCEPTABILITY |  |
| Is the option acceptable to key stakeholders? | Probably yes |
| FEASABILITY |  |
| Is the option feasible to implement? | Probably yes |

**Overall conclusions:**

| Balance of consequences | | | | |
| --- | --- | --- | --- | --- |
| Undesirable consequences  *clearly outweigh*  desirable consequences in most settings  ☐ | Undesirable consequences *probably outweigh*  desirable consequences in most settings  ☐ | The balance between  desirable and undesirable consequences  *is closely balanced or uncertain*  ☐ | **Desirable consequences  *probably outweigh* undesirable consequences in most settings**  ■ | Desirable consequences  *clearly outweigh*  undesirable consequences in most settings  ☐ |

**Recommendation:**

| Type of recommendation – Events with altitude or pressure differences | | | |
| --- | --- | --- | --- |
| We strongly believe not to  ☐ | We believe not to  ☐ | **We believe that**  ■ | We strongly believe that  ☐ |
| Recommendation (text) | **We believe that clinically stable children with cancer without neutropenia (i.e. neutrophil count <0.5x10^9^/L) or thrombocytopenia (i.e. platelet count <50x10^9^/L) can perform events with altitude or pressure differences, such as going on a plane or scuba diving in agreement with their treating physician. (WEAK recommendation, EXPERT evidence)** | | |
| Justification | No evidence in pediatric oncology patients was found. Therefore, the recommendation is based on expert opinions.  The guideline panel believes that children with cancer can perform high or low altitude events such as going on a plane or scuba diving. We believe that children in a stable phase of their treatment without severe neutropenia or thrombocytopenia, should be allowed to perform these activities. We see no obvious reasons why these events with altitude or pressure differences should be contraindicated in stable children. | | |
| Subgroup considerations | This recommendation only accounts for clinically stable children with cancer without neutropenia (i.e. neutrophil count <0.5x10^9^/L) or thrombocytopenia (i.e. platelet count <50x10^9^/L). | | |
| Implementation considerations | Note, this should always be a careful consideration for the child as an individual, and therefore this always needs to be discussed and be allowed by the treating physician. | | |
| Monitoring and evaluation | Not applicable. | | |
| Research priorities | Not applicable. | | |

**Evidence to decision framework 7: Hygiene (general)

Recommendation:**

| Type of recommendation – Hygiene (general) | |
| --- | --- |
| Recommendation (text) | **Proper hand hygiene should be performed by patients, caregivers and medical personnel. (STRONG recommendation, GOOD PRACTICE STATEMENT)** |
| Justification | No evidence in pediatric oncology patients was found for this clinical question. The recommendation from the ASCO and IDSA (5) guideline was used, and expert opinions were discussed.  The guideline panel strongly agrees that proper hand hygiene is very important for patients, caregivers and medical personnel. We therefore agreed to a recommendation in line with the recommendation from the ASCO and IDSA guideline. There is no clear evidence that supports this recommendation, but it is supported by a combination of general logic, common sense and expert opinions. Therefore, this recommendation is a ‘best practice statement’. |
| Subgroup considerations | Not applicable. |
| Implementation considerations | Not applicable. |
| Monitoring and evaluation | Not applicable. |
| Research priorities | Not applicable. |

**Evidence to decision framework 8: Hygiene (personal)**

|  | Criteria | Judgements | Research evidence | Additional considerations |
| --- | --- | --- | --- | --- |
| PROBLEM | Is the problem a priority? | ☐ No  ☐ Probably no  ☐ Uncertain  ☐ Probably yes  ■ **Yes** | Improving quality of life has become increasingly important in care for children with cancer. Due to improved survival rates there is an increased focus on morbidity and adverse effects of anti-cancer treatment (1, 2). To prevent adverse health problems, such as infections and bleeding, restrictions in daily life have been defined for children with cancer related to school attendance, travelling with public transport, pets, hygiene measures and swimming (3). However, these social restrictions can severely impair the quality of life of these children (4, 5).  Within the Netherlands, there is large variation in current supportive care practices, including social restrictions (6). The majority of these recommendations regarding social restrictions for children with cancer are not evidence-based. Guidance is necessary in order to provide the best possible care for these children, balancing cautiousness and restrictiveness. |  |
| BENEFITS AND HARMS | What is the overall certainty of this evidence? | ■ **No included studies**  ☐ Very low  ☐ Low  ☐ Moderate  ☐ High | No evidence in pediatric oncology patients was found for this clinical question. Therefore, the recommendation is based on expert opinions.  The guideline panel agrees that basic hygiene measures are sufficient for children with cancer. There is no need for washing clothes separately or extra, cleaning the house in extreme form, or renewing clothes multiple times a day. We believe that as long as the household is cleaned in a normal way, this would be a sufficient amount of hygiene measures. There is no need to intensify any of this personal hygiene measures such as cleaning the house or doing laundry. |  |
|  | Is there important uncertainty about how much people value the main outcomes? | ☐ Important uncertainty or variability  ☐ Possibly important uncertainty or variability  ☐ Probably no important uncertainty or variability  ■ **No important uncertainty or variability**  ☐ No known undesirable outcomes |  |  |
|  | Are the desirable anticipated effects large? | ☐ No  ☐ Probably no  ☐ Uncertain  ☐ Probably yes  ■ **Yes**  ☐ Varies |  |  |
|  | Are the undesirable anticipated effects small? | ☐ No  ■**Probably no**  ☐ Uncertain  ☐ Probably yes  ☐ Yes  ☐ Varies |  |  |
|  | Are the desirable effects large relative to undesirable effects? | ☐ No  ☐ Probably no  ☐Uncertain  ■ **Probably yes**  ☐ Yes  ☐ Varies |  |  |
| RESOURCE USE | Are the resources required small? | ☐ No  ☐ Probably no  ☐ Uncertain  ☐ Probably yes  ■ **Yes**  ☐ Varies |  |  |
|  | Is the incremental cost small relative to the net benefits? | ☐ No  ☐ Probably no  ☐ Uncertain  ☐ Probably yes  ■ **Yes**  ☐ Varies |  |  |
| EQUITY | What would be the impact on health inequities? | ☐ Increased  ☐ Probably increased  ■ **Uncertain**  ☐ Probably reduced  ☐ Reduced  ☐ Varies |  | The panel expects that this daily life restriction will not have any effect on health inequities |
| ACCEPTABILITY | Is the option acceptable to key stakeholders? | ☐ No  ☐ Probably no  ☐ Uncertain  ☐ Probably yes  ■ **Yes**  ☐ Varies |  |  |
| FEASIBILITY | Is the option feasible to implement? | ☐ No  ☐ Probably no  ☐ Uncertain  ☐ Probably yes  ■**Yes**  ☐ Varies |  | This accounts for feasibility in the Netherlands. For low income countries, this option may be less feasible to implement. |

**Conclusion EtD:**

| PROBLEM |  |
| --- | --- |
| Is the problem a priority? | Yes |
| BENEFITS AND HARMS |  |
| What is the overall certainty of this evidence? | No included studies |
| Is there important uncertainty about how much people value the main outcomes? | No important uncertainty or variability |
| Are the desirable anticipated effects large? | Yes |
| Are the undesirable anticipated effects small? | Probably no |
| Are the desirable effects large relative to undesirable effects? | Probably yes |
| RESOURCE USE |  |
| Are the resources required small? | Yes |
| Is the incremental cost small relative to the net benefits? | Yes |
| EQUITY |  |
| What would be the impact on health inequities? | Uncertain |
| ACCEPTABILITY |  |
| Is the option acceptable to key stakeholders? | Yes |
| FEASABILITY |  |
| Is the option feasible to implement? | Yes |

**Overall conclusions:**

| Balance of consequences | | | | |
| --- | --- | --- | --- | --- |
| Undesirable consequences  *clearly outweigh*  desirable consequences in most settings  ☐ | Undesirable consequences *probably outweigh*  desirable consequences in most settings  ☐ | The balance between  desirable and undesirable consequences  *is closely balanced or uncertain*  ☐ | Desirable consequences  *probably outweigh* undesirable consequences in most settings  ☐ | **Desirable consequences  *clearly outweigh*  undesirable consequences in most settings**  ■ |

**Recommendation:**

| Type of recommendation – Hygiene (general) | | | |
| --- | --- | --- | --- |
| We strongly believe not to  ☐ | We believe not to  ☐ | We believe that  ☐ | **We strongly believe that**  ■ |
| Recommendation (text) | **We strongly believe that regular personal hygiene (regarding doing laundry, cleaning, renewing clothes) is sufficient for children with cancer and their households. (STRONG recommendation, EXPERT evidence)** | | |
| Justification | No evidence in pediatric oncology patients was found for this clinical question. Therefore, the recommendation is based on expert opinions. The guideline panel agrees that basic hygiene measures are sufficient for children with cancer. There is no need for washing clothes separately or extra, cleaning the house in extreme form, or renewing clothes multiple times a day. We believe that as long as the household is cleaned in a normal way, this would be a sufficient amount of hygiene measures. There is no need to intensify any of this personal hygiene measures such as cleaning the house or doing laundry. | | |
| Subgroup considerations | Not applicable. | | |
| Implementation considerations | Not applicable. | | |
| Monitoring and evaluation | Not applicable. | | |
| Research priorities | Not applicable. | | |

**Evidence to decision framework 9: Pets, zoo and farm**

|  | Criteria | Judgements | Research evidence | Additional considerations |
| --- | --- | --- | --- | --- |
| PROBLEM | Is the problem a priority? | ☐ No  ☐ Probably no  ☐ Uncertain  ☐ Probably yes  ■ **Yes** | Improving quality of life has become increasingly important in care for children with cancer. Due to improved survival rates there is an increased focus on morbidity and adverse effects of anti-cancer treatment (1, 2). To prevent adverse health problems, such as infections and bleeding, restrictions in daily life have been defined for children with cancer related to school attendance, travelling with public transport, pets, hygiene measures and swimming (3). However, these social restrictions can severely impair the quality of life of these children (4, 5).  Within the Netherlands, there is large variation in current supportive care practices, including social restrictions (6). The majority of these recommendations regarding social restrictions for children with cancer are not evidence-based. Guidance is necessary in order to provide the best possible care for these children, balancing cautiousness and restrictiveness. |  |
| BENEFITS AND HARMS | What is the overall certainty of this evidence? | ☐ No included studies  ■ **Very low**  ☐ Low  ☐ Moderate  ☐ High | \| Outcome \| Relative importance \| Quality of evidence \| \| \| --- \| --- \| --- \| --- \| \| Number of infections \| Critical \| Very low \|   Summary of findings:  1. Number of infections  1A1. When the results were adjusted for gender, age, weight groups, risk stratification and antibiotic prophylaxis, restriction of pets was **not** significantly associated with decreased risk of fever of unknown origin. IRR 0.99, 95% CI 0.95-1.03, p=0.59. 1A2. For adjusted results (see above), restriction of pets was **not** significantly associated with decreased risk of bacteremia. IRR 0.99, 95% CI 0.94-1.05, p=0.75. 1A3. For adjusted results (see above), restriction of pets was **not** significantly associated with decreased risk of pneumonia. IRR 0.91, 95% CI 0.82-1.02, p=0.11. 1A4. For adjusted results (see above), restriction of pets was **not** significantly associated with decreased risk of gastroenteritis. IRR 1.05, 95% CI 0.95-1.17, p=0.316. |  |
|  | Is there important uncertainty about how much people value the main outcomes? | ☐ Important uncertainty or variability  ☐ Possibly important uncertainty or variability  ☐ Probably no important uncertainty or variability  ■ **No important uncertainty or variability**  ☐ No known undesirable outcomes |  |  |
|  | Are the desirable anticipated effects large? | ☐ No  ☐ Probably no  ☐ Uncertain  ☐ Probably yes  ■ **Yes**  ☐ Varies |  | For pets the desirable anticipated effects are large, for visiting the zoo or farm probably yes. |
|  | Are the undesirable anticipated effects small? | ☐ No  ■ **Probably no**  ☐ Uncertain  ☐ Probably yes  ☐ Yes  ☐ Varies |  |  |
|  | Are the desirable effects large relative to undesirable effects? | ☐ No  ☐ Probably no  ■ **Uncertain**  ☐ Probably yes  ☐ Yes  ☐ Varies |  |  |
| RESOURCE USE | Are the resources required small? | ☐ No  ☐ Probably no  ☐ Uncertain  ☐ Probably yes  ■ **Yes**  ☐ Varies |  |  |
|  | Is the incremental cost small relative to the net benefits? | ☐ No  ☐ Probably no  ☐ Uncertain  ■ **Probably yes**  ☐ Yes  ☐ Varies |  |  |
| EQUITY | What would be the impact on health inequities? | ☐ Increased  ☐ Probably increased  ■ **Uncertain**  ☐ Probably reduced  ☐ Reduced  ☐ Varies |  | The panel expects that this daily life restriction will not have any effect on health inequities |
| ACCEPTABILITY | Is the option acceptable to key stakeholders? | ☐ No  ☐ Probably no  ☐ Uncertain  ☐ Probably yes  ■ **Yes**  ☐ Varies |  |  |
| FEASIBILITY | Is the option feasible to implement? | ☐ No  ☐ Probably no  ☐ Uncertain  ☐ Probably yes  ■ **Yes**  ☐ Varies |  |  |

**Conclusion EtD:**

| PROBLEM |  |
| --- | --- |
| Is the problem a priority? | Yes |
| BENEFITS AND HARMS |  |
| What is the overall certainty of this evidence? | Very low quality |
| Is there important uncertainty about how much people value the main outcomes? | No important uncertainty or variability |
| Are the desirable anticipated effects large? | Yes |
| Are the undesirable anticipated effects small? | Probably no |
| Are the desirable effects large relative to undesirable effects? | Uncertain |
| RESOURCE USE |  |
| Are the resources required small? | Yes |
| Is the incremental cost small relative to the net benefits? | Probably yes |
| EQUITY |  |
| What would be the impact on health inequities? | Uncertain |
| ACCEPTABILITY |  |
| Is the option acceptable to key stakeholders? | Yes |
| FEASABILITY |  |
| Is the option feasible to implement? | Yes |

**Overall conclusions:**

| Balance of consequences | | | | |
| --- | --- | --- | --- | --- |
| Undesirable consequences  *clearly outweigh*  desirable consequences in most settings  ☐ | Undesirable consequences *probably outweigh*  desirable consequences in most settings  ☐ | The balance between  desirable and undesirable consequences  *is closely balanced or uncertain*  ☐ | **Desirable consequences  *probably outweigh* undesirable consequences in most settings**  ■ | Desirable consequences  *clearly outweigh*  undesirable consequences in most settings  ☐ |

**Recommendation:**

| Type of recommendation – Pets, zoo and farm | | | |
| --- | --- | --- | --- |
| We recommend against  offering this option  ☐ | We suggest not offering  this option  ☐ | **We suggest offering**  **this option**  ■ | We recommend offering  this option  ☐ |
| Recommendation (text) | **We suggest allowing to keep domestic pets in the households of children with cancer. (WEAK recommendation, VERY LOW quality of evidence)**  **We believe that children with cancer are allowed to go to the zoo or visit a farm. (WEAK recommendation, EXPERT evidence)**  **We believe that children with cancer should not clean the litterbox or cage of their domestic pets. (WEAK recommendation, EXPERT evidence)** | | |
| Justification | One study in pediatric oncology patients was included for this clinical question (11), in which, restriction of pets at home was not significantly associated with a decreased risk of any type of infection. However, this study described the restriction advice that was given by the doctors, and does not report on the actual adherence of the patients. This study is retrospective and may not show the exact restriction of pets of the patients. However, there was no effect found of the restriction, which is supported by our expert opinions.  The guideline panel agreed that any restriction in pets at home is not necessary. If children consider regular their hand hygiene after playing with or touching their pet, we see no reason why any other form of restriction should be advised. We believe risk of infection from a pet is minimal, considering adequate hand hygiene, and that the quality of life would substantially decrease if there would be any form of restriction regarding the pets. Under these conditions, the guideline panel feels that children with cancer can keep and play with their pets.  We also believe that children with cancer should be allowed to visit the zoo or farm. If the children remain at distance from the animals, we see no problems regarding infectious risks. If the children, for example on a farm, touch the pets or feed them, they should again consider their hand hygiene. Under these conditions, the guideline panel feels that children with cancer can safely visit the zoo or farm.   However, we do suggest that children with cancer do not clean the cages and/or litter boxes of the pets. We consider the infectious risk higher for these tasks, and it can easily – with no to minimal decrease in quality of life – be avoided by children with cancer.  In summary, taking adequate hand hygiene into consideration, we believe children with cancer can keep and play with their pets, and safely visit the zoo or farm, as the risk of infection would be very minimal and the quality of life would decrease with any form of restriction. | | |
| Subgroup considerations | Not applicable. | | |
| Implementation considerations | Additionally, we also suggest that the pets of these children are seen by a veterinarian and that they are in good health. | | |
| Monitoring and evaluation | Not applicable. | | |
| Research priorities | Not applicable. | | |

**Evidence to decision framework 10: Public transport**

|  | Criteria | Judgements | Research evidence | Additional considerations |
| --- | --- | --- | --- | --- |
| PROBLEM | Is the problem a priority? | ☐ No  ☐ Probably no  ☐ Uncertain  ☐ Probably yes  ■ **Yes** | Improving quality of life has become increasingly important in care for children with cancer. Due to improved survival rates there is an increased focus on morbidity and adverse effects of anti-cancer treatment (1, 2). To prevent adverse health problems, such as infections and bleeding, restrictions in daily life have been defined for children with cancer related to school attendance, travelling with public transport, pets, hygiene measures and swimming (3). However, these social restrictions can severely impair the quality of life of these children (4, 5).  Within the Netherlands, there is large variation in current supportive care practices, including social restrictions (6). The majority of these recommendations regarding social restrictions for children with cancer are not evidence-based. Guidance is necessary in order to provide the best possible care for these children, balancing cautiousness and restrictiveness. |  |
| BENEFITS AND HARMS | What is the overall certainty of this evidence? | ■ **No included studies**  ☐ Very low  ☐ Low  ☐ Moderate  ☐ High | No evidence in pediatric oncology patients was found for this clinical question. Therefore, the recommendation is based on expert opinions.  The guideline panel agrees that basic hygiene measures are sufficient for children with cancer. We believe there is no need to avoid public transport as long as basic hygiene measures such as hand hygiene are performed. Then, we believe the risk of infection remains minimal.   The guideline panel does feel that there is an exception for children with cancer and neutropenia, who should avoid the public transport or crowded places in winter months. In these months, there is a higher incidence of viral infections and thereby they have a higher chance of getting infected. As the potential consequences of a viral infection can be big (for example, hospital admission because of fever, delay of chemotherapy or the need for antiviral medication), we believe the public transport should be avoided in these ‘high risk’ months (defined by the guideline panel as September to March). | It is difficult to precisely describe the months with a higher incidence of viral infections in general. Therefore, it is for each caregiver to individually decide what those high risk months are in your country. |
|  | Is there important uncertainty about how much people value the main outcomes? | ☐ Important uncertainty or variability  ☐ Possibly important uncertainty or variability  ☐ Probably no important uncertainty or variability  ■ **No important uncertainty or variability**  ☐ No known undesirable outcomes |  |  |
|  | Are the desirable anticipated effects large? | ☐ No  ☐ Probably no  ☐ Uncertain  ■ **Probably yes**  ☐ Yes  ☐ Varies |  |  |
|  | Are the undesirable anticipated effects small? | ☐ No  ■ **Probably no**  ☐ Uncertain  ☐ Probably yes  ☐ Yes  ☐ Varies |  |  |
|  | Are the desirable effects large relative to undesirable effects? | ☐ No  ☐ Probably no  ■**Uncertain**  ☐ Probably yes  ☐ Yes  ☐ Varies |  |  |
| RESOURCE USE | Are the resources required small? | ☐ No  ☐ Probably no  ☐ Uncertain  ■**Probably yes**  ☐ Yes  ☐ Varies |  |  |
|  | Is the incremental cost small relative to the net benefits? | ☐ No  ☐ Probably no  ■ **Uncertain**  ☐ Probably yes  ☐ Yes  ☐ Varies |  |  |
| EQUITY | What would be the impact on health inequities? | ☐ Increased  ☐ Probably increased  ■ **Uncertain**  ☐ Probably reduced  ☐ Reduced  ☐ Varies |  | The panel expects that this daily life restriction will not have any effect on health inequities.   Some children might be depended on public transport, we hope that an arrangement can be made for these children specifically. |
| ACCEPTABILITY | Is the option acceptable to key stakeholders? | ☐ No  ☐ Probably no  ☐ Uncertain  ■ **Probably yes**  ☐ Yes  ☐ Varies |  |  |
| FEASIBILITY | Is the option feasible to implement? | ☐ No  ☐ Probably no  ☐ Uncertain  ■ **Probably yes**  ☐Yes  ☐ Varies |  |  |

**Conclusion EtD:**

| PROBLEM |  |
| --- | --- |
| Is the problem a priority? | Yes |
| BENEFITS AND HARMS |  |
| What is the overall certainty of this evidence? | No included studies |
| Is there important uncertainty about how much people value the main outcomes? | No important uncertainty or variability |
| Are the desirable anticipated effects large? | Probably yes |
| Are the undesirable anticipated effects small? | Probably no |
| Are the desirable effects large relative to undesirable effects? | Uncertain |
| RESOURCE USE |  |
| Are the resources required small? | Probably yes |
| Is the incremental cost small relative to the net benefits? | Uncertain |
| EQUITY |  |
| What would be the impact on health inequities? | Uncertain |
| ACCEPTABILITY |  |
| Is the option acceptable to key stakeholders? | Probably yes |
| FEASABILITY |  |
| Is the option feasible to implement? | Probably yes |

**Overall conclusions:**

| Balance of consequences | | | | |
| --- | --- | --- | --- | --- |
| Undesirable consequences  *clearly outweigh*  desirable consequences in most settings  ☐ | Undesirable consequences *probably outweigh*  desirable consequences in most settings  ☐ | The balance between  desirable and undesirable consequences  *is closely balanced or uncertain*  ☐ | **Desirable consequences  *probably outweigh* undesirable consequences in most settings**  ■ | Desirable consequences  *clearly outweigh*  undesirable consequences in most settings  ☐ |

**Recommendation:**

| Type of recommendation – Public transport | | | |
| --- | --- | --- | --- |
| We strongly believe not to  ☐ | We believe not to  ☐ | **We believe that**  ■ | We strongly believe that  ☐ |
| Recommendation (text) | **We believe that children with cancer are allowed to use public transport or visit crowded places (i.e. big events such as visiting a concert or theater). (WEAK recommendation, EXPERT evidence)** | | |
| Justification | No evidence in pediatric oncology patients was found for this clinical question. Therefore, the recommendation is based on expert opinions.  The guideline panel agrees that basic hygiene measures are sufficient for children with cancer. We believe there is no need to avoid public transport as long as basic hygiene measures such as hand hygiene are performed. Then, we believe the risk of infection remains minimal. | | |
| Subgroup considerations | The guideline panel does feel that there is an exception for children with cancer and neutropenia, who should avoid the public transport or crowded places in winter months. In these months, there is a higher incidence of viral infections and thereby they have a higher chance of getting infected. As the potential consequences of a viral infection can be big (for example, hospital admission because of fever, delay of chemotherapy or the need for antiviral medication), we believe the public transport should be avoided in these ‘high risk’ months. | | |
| Implementation considerations | The guideline panel emphasizes the basic hygiene measures for children with cancer | | |
| Monitoring and evaluation | Not applicable. | | |
| Research priorities | Not applicable. | | |

**Evidence to decision framework 11: School and kindergarten**

|  | Criteria | Judgements | Research evidence | Additional considerations |
| --- | --- | --- | --- | --- |
| PROBLEM | Is the problem a priority? | ☐ No  ☐ Probably no  ☐ Uncertain  ☐ Probably yes  ■ **Yes** | Improving quality of life has become increasingly important in care for children with cancer. Due to improved survival rates there is an increased focus on morbidity and adverse effects of anti-cancer treatment (1, 2). To prevent adverse health problems, such as infections and bleeding, restrictions in daily life have been defined for children with cancer related to school attendance, travelling with public transport, pets, hygiene measures and swimming (3). However, these social restrictions can severely impair the quality of life of these children (4, 5).  Within the Netherlands, there is large variation in current supportive care practices, including social restrictions (6). The majority of these recommendations regarding social restrictions for children with cancer are not evidence-based. Guidance is necessary in order to provide the best possible care for these children, balancing cautiousness and restrictiveness. |  |
| BENEFITS AND HARMS | What is the overall certainty of this evidence? | ☐ No included studies  ■ **Very low**  ☐ Low  ☐ Moderate  ☐ High | \| Outcome \| Relative importance \| Quality of evidence \| \| \| --- \| --- \| --- \| --- \| \| Number of infections \| Critical \| Very low \|   Summary of findings:  1. Number of infections 1A1. When the results were adjusted for gender, age, weight groups, risk stratification and antibiotic prophylaxis, restriction of social contact was **not** significantly associated with decreased risk of fever of unknown origin. IRR 0.99, 95% CI 0.92-1.08, p=0.9. 1A2. For adjusted results (see above), restriction of social contact was **not** significantly associated with decreased risk of bacteremia. IRR 1.15, 95% CI 0.99-1.33, p=0.066. 1A3. For adjusted results (see above), restriction of social contact was **not** significantly associated with decreased risk of pneumonia. IRR 0.99, 95% CI 0.77-1.28, p=0.96. 1A4. For adjusted results (see above), restriction of pets was **not** significantly associated with decreased risk of gastroenteritis. IRR 0.94, 95% CI 0.73-1.21, p=0.63. |  |
|  | Is there important uncertainty about how much people value the main outcomes? | ☐ Important uncertainty or variability  ☐ Possibly important uncertainty or variability  ☐ Probably no important uncertainty or variability  ■ **No important uncertainty or variability**  ☐ No known undesirable outcomes |  |  |
|  | Are the desirable anticipated effects large? | ☐ No  ☐ Probably no  ☐ Uncertain  ☐ Probably yes  ■ **Yes**  ☐ Varies |  |  |
|  | Are the undesirable anticipated effects small? | ☐ No  ■ **Probably no**  ☐ Uncertain  ☐ Probably yes  ☐ Yes  ☐ Varies |  |  |
|  | Are the desirable effects large relative to undesirable effects? | ☐ No  ☐ Probably no  ☐Uncertain  ■ **Probably yes**  ☐ Yes  ☐ Varies |  |  |
| RESOURCE USE | Are the resources required small? | ☐ No  ☐ Probably no  ☐ Uncertain  ☐ Probably yes  ■ **Yes**  ☐ Varies |  |  |
|  | Is the incremental cost small relative to the net benefits? | ☐ No  ☐ Probably no  ☐ Uncertain  ☐ Probably yes  ■ **Yes**  ☐ Varies |  |  |
| EQUITY | What would be the impact on health inequities? | ☐ Increased  ☐ Probably increased  ■ **Uncertain**  ☐ Probably reduced  ☐ Reduced  ☐ Varies |  | The panel expects that this daily life restriction will not have any effect on health inequities. |
| ACCEPTABILITY | Is the option acceptable to key stakeholders? | ☐ No  ☐ Probably no  ☐ Uncertain  ☐ Probably yes  ■ **Yes**  ☐ Varies |  |  |
| FEASIBILITY | Is the option feasible to implement? | ☐ No  ☐ Probably no  ☐ Uncertain  ■ **Probably yes**  ☐Yes  ☐ Varies |  |  |

**Conclusion EtD:**

| PROBLEM |  |
| --- | --- |
| Is the problem a priority? | Yes |
| BENEFITS AND HARMS |  |
| What is the overall certainty of this evidence? | Very low quality |
| Is there important uncertainty about how much people value the main outcomes? | No important uncertainty or variability |
| Are the desirable anticipated effects large? | Yes |
| Are the undesirable anticipated effects small? | Probably no |
| Are the desirable effects large relative to undesirable effects? | Probably yes |
| RESOURCE USE |  |
| Are the resources required small? | Yes |
| Is the incremental cost small relative to the net benefits? | Yes |
| EQUITY |  |
| What would be the impact on health inequities? | Uncertain |
| ACCEPTABILITY |  |
| Is the option acceptable to key stakeholders? | Yes |
| FEASABILITY |  |
| Is the option feasible to implement? | Probably yes |

**Overall conclusions:**

| Balance of consequences | | | | |
| --- | --- | --- | --- | --- |
| Undesirable consequences  *clearly outweigh*  desirable consequences in most settings  ☐ | Undesirable consequences *probably outweigh*  desirable consequences in most settings  ☐ | The balance between  desirable and undesirable consequences  *is closely balanced or uncertain*  ☐ | Desirable consequences  *probably outweigh* undesirable consequences in most settings  ☐ | **Desirable consequences  *clearly outweigh*  undesirable consequences in most settings**  ■ |

**Recommendation:**

| Type of recommendation – School and kindergarten | | | |
| --- | --- | --- | --- |
| We recommend against  offering this option  ☐ | We suggest not offering  this option  ☐ | We suggest offering  this option  ☐ | **We recommend offering**  **this option**  ■ |
| Recommendation (text) | **We recommend allowing children with cancer to attend school or kindergarten irrespective of neutropenia (unless someone in their class or group has a contagious disease with potential severe consequences, e.g. varicella zoster). (STRONG recommendation, VERY LOW quality evidence)** | | |
| Justification | One study in pediatric oncology patients was included for this clinical question. In conclusion, restriction of social contact was not significantly associated with a decreased risk of any type of infection. However, this study described the restriction advice that was given by the doctors, and does not report on the actual adherence of the patients. This study is retrospective and may not show the exact social restrictions of the patients, but it does show an effect of the social restriction, which is supported by our expert opinions.  In line with the above mentioned study, the guideline panel feels that any restriction in daily life such as restriction in school or kindergarten is not necessary. If children carefully consider their hand hygiene during the day, we see no reason why any other form of social restriction should be advised.  The guideline panel recognizes that the risk of infection at schools or kindergarten may be a concern to parents. However, we agree that going to school or kindergarten increases the quality of life of these children, as their social and cognitive development, in such a way that it outweighs the harms. Going to school is very important for the development of any child, also for children with cancer. It also has an important social aspect of seeing their friends at school and continuing with their life in the best possible way.  However, we recognize that if a lot of children in the class or at kindergarten are sick, parents would choose to keep their children at home. We strongly suggest that children stay at home when someone in their class or group has a contagious disease such as chickenpox. This only accounts for children who did not had chickenpox before or have no antibody titer. If there is an infectious disease in the class or at kindergarten, the guideline panel suggests that this will then be discussed by the treating physician for the specific patient. In summary, taking adequate hand hygiene into consideration, we believe children with cancer should attend school or kindergarten (unless someone in their class or group has an infectious disease such as chickenpox), as the risk of infection would be minimal and the quality of life would decrease with any form of restriction. | | |
| Subgroup considerations | We strongly suggest that children stay at home when someone in their class or group has a contagious disease with potential severe consequences, e.g. varicella zoster. If this is the case, the guideline panel suggests that this will then be discussed by the treating physician for the specific patient to discuss the benefits and harms of going to school or kindergarten in that specific case. | | |
| Implementation considerations | Not applicable. | | |
| Monitoring and evaluation | Not applicable. | | |
| Research priorities | The guideline panel feels that school is very important for children in general, and that this should be stimulated as much as possible. For us, this is a research priority in the upcoming years to make sure children can go to school as much (and as safe) as possible. | | |

**Evidence to decision framework 12: Sports and high-velocity events**

|  | Criteria | Judgements | Research evidence | Additional considerations |
| --- | --- | --- | --- | --- |
| PROBLEM | Is the problem a priority? | ☐ No  ☐ Probably no  ☐ Uncertain  ☐ Probably yes  ■ **Yes** | Improving quality of life has become increasingly important in care for children with cancer. Due to improved survival rates there is an increased focus on morbidity and adverse effects of anti-cancer treatment (1, 2). To prevent adverse health problems, such as infections and bleeding, restrictions in daily life have been defined for children with cancer related to school attendance, travelling with public transport, pets, hygiene measures and swimming (3). However, these social restrictions can severely impair the quality of life of these children (4, 5).  Within the Netherlands, there is large variation in current supportive care practices, including social restrictions (6). The majority of these recommendations regarding social restrictions for children with cancer are not evidence-based. Guidance is necessary in order to provide the best possible care for these children, balancing cautiousness and restrictiveness. |  |
| BENEFITS AND HARMS | What is the overall certainty of this evidence? | ■**No included studies**  ☐ Very low  ☐ Low  ☐ Moderate  ☐ High | No evidence in pediatric oncology patients was found for this clinical question. Therefore, the recommendation is based on expert opinions.  Firstly, the guideline panel strongly believes that children with cancer are allowed (and should be encouraged) to exercise and perform sports. Besides the reason of thrombocytopenia or other individual reasons, it is always encouraged for children to perform sports and other activities. This greatly benefits their physical state, but also their quality of life. | However, the guideline panel feels that an exception needs to be made for children with thrombocytopenia (i.e. thrombocytes <50x10^9^/L). In some types of activities, such as contact sports, high-impact or high-velocity events, events with risk of falling, the risk of bleeding is just too big when a child has thrombocytopenia. Therefore, these activities should be avoided in the event of thrombocytopenia. We suggest encouraging these children to perform activities that are safe, to ensure the positive consequences of performing activities and sports. |
|  | Is there important uncertainty about how much people value the main outcomes? | ☐ Important uncertainty or variability  ☐ Possibly important uncertainty or variability  ☐ Probably no important uncertainty or variability  ■ **No important uncertainty or variability**  ☐ No known undesirable outcomes |  |  |
|  | Are the desirable anticipated effects large? | ☐ No  ☐ Probably no  ☐ Uncertain  ■ **Probably yes** *(high-velocity events)*  ■ **Yes** *(sports)*  ☐ Varies |  | Yes for sports, probably yes for high-velocity events. |
|  | Are the undesirable anticipated effects small? | ☐ No  ■ **Probably no**  ☐ Uncertain  ☐ Probably yes  ☐ Yes  ☐ Varies |  |  |
|  | Are the desirable effects large relative to undesirable effects? | ☐ No  ☐ Probably no  ■**Uncertain**  ☐ Probably yes  ☐ Yes  ☐ Varies |  |  |
| RESOURCE USE | Are the resources required small? | ☐ No  ☐ Probably no  ☐ Uncertain  ☐ Probably yes  ■ **Yes**  ☐ Varies |  |  |
|  | Is the incremental cost small relative to the net benefits? | ☐ No  ☐ Probably no  ■ **Uncertain**  ☐ Probably yes  ☐ Yes  ☐ Varies |  |  |
| EQUITY | What would be the impact on health inequities? | ☐ Increased  ☐ Probably increased  ■ **Uncertain**  ☐ Probably reduced  ☐ Reduced  ☐ Varies |  |  |
| ACCEPTABILITY | Is the option acceptable to key stakeholders? | ☐ No  ☐ Probably no  ☐ Uncertain  ☐ Probably yes  ■ **Yes**  ☐ Varies |  |  |
| FEASIBILITY | Is the option feasible to implement? | ☐ No  ☐ Probably no  ☐ Uncertain  ■ **Probably yes**  ☐Yes  ☐ Varies |  |  |

**Conclusion EtD:**

| PROBLEM |  |
| --- | --- |
| Is the problem a priority? | Yes |
| BENEFITS AND HARMS |  |
| What is the overall certainty of this evidence? | No included studies |
| Is there important uncertainty about how much people value the main outcomes? | No important uncertainty or variability |
| Are the desirable anticipated effects large? | Yes/Probably yes |
| Are the undesirable anticipated effects small? | Probably no |
| Are the desirable effects large relative to undesirable effects? | Uncertain |
| RESOURCE USE |  |
| Are the resources required small? | Yes |
| Is the incremental cost small relative to the net benefits? | Uncertain |
| EQUITY |  |
| What would be the impact on health inequities? | Uncertain |
| ACCEPTABILITY |  |
| Is the option acceptable to key stakeholders? | Yes |
| FEASABILITY |  |
| Is the option feasible to implement? | Probably yes |

**Overall conclusions:**

| Balance of consequences | | | | |
| --- | --- | --- | --- | --- |
| Undesirable consequences  *clearly outweigh*  desirable consequences in most settings  ☐ | Undesirable consequences *probably outweigh*  desirable consequences in most settings  ☐ | The balance between  desirable and undesirable consequences  *is closely balanced or uncertain*  ☐ | Desirable consequences  *probably outweigh* undesirable consequences in most settings  ☐ | **Desirable consequences  *clearly outweigh*  undesirable consequences in most settings**  ■ |

**Recommendation:**

| Type of recommendation – Sports and high-velocity events | | | |
| --- | --- | --- | --- |
| We strongly believe not to  ☐ | We believe not to  ☐ | We believe that  ☐ | **We strongly believe that**  ■ |
| Recommendation (text) | **We strongly believe that children with cancer should be encouraged to exercise and perform sports. (STRONG recommendation, EXPERT evidence)**  **We believe that children with cancer with thrombocytopenia (i.e. platelet count <50x10^9^/L) should not perform events with increased risk of bleeding (contact sports, high-impact or high-velocity events, events with risk of falling). (WEAK recommendation, EXPERT evidence)** | | |
| Justification | No evidence in pediatric oncology patients was found for this clinical question. Therefore, the recommendation is based on expert opinions. Firstly, the guideline panel strongly believes that children with cancer are allowed (and should be encouraged) to exercise and perform sports. Besides the exception of thrombocytopenia or other individual reasons, it is always encouraged for children to perform sports and other activities. This greatly benefits their physical state, but also their quality of life. | | |
| Subgroup considerations | The guideline panel feels that an exception needs to be made for children with thrombocytopenia (i.e. thrombocytes <50x10^9^/L). In some types of activities, such as contact sports, high-impact or high-velocity events, events with risk of falling, the risk of bleeding is just too big when a child has thrombocytopenia. Therefore, these activities should be avoided in the event of thrombocytopenia. We suggest encouraging these children to perform activities that are safe, to ensure the positive consequences of performing activities and sports. | | |
| Implementation considerations | Not applicable. | | |
| Monitoring and evaluation | Not applicable. | | |
| Research priorities | Not applicable. | | |

**Evidence to decision framework 13: Swimming**

|  | Criteria | Judgements | Research evidence | Additional considerations |
| --- | --- | --- | --- | --- |
| PROBLEM | Is the problem a priority? | ☐ No  ☐ Probably no  ☐ Uncertain  ☐ Probably yes  ■ **Yes** | Improving quality of life has become increasingly important in care for children with cancer. Due to improved survival rates there is an increased focus on morbidity and adverse effects of anti-cancer treatment (1, 2). To prevent adverse health problems, such as infections and bleeding, restrictions in daily life have been defined for children with cancer related to school attendance, travelling with public transport, pets, hygiene measures and swimming (3). However, these social restrictions can severely impair the quality of life of these children (4, 5).  Within the Netherlands, there is large variation in current supportive care practices, including social restrictions (6). The majority of these recommendations regarding social restrictions for children with cancer are not evidence-based. Guidance is necessary in order to provide the best possible care for these children, balancing cautiousness and restrictiveness. |  |
| BENEFITS AND HARMS | What is the overall certainty of this evidence? | ☐ No included studies  ■ **Very low**  ☐ Low  ☐ Moderate  ☐ High | \| Outcome \| Relative importance \| Quality of evidence \| \| \| --- \| --- \| --- \| --- \| \| Number of infections \| Critical \| Very low \|   Summary of findings:  1.1. Number of infections “Swimming”  1.1A. Total of infections: 34 in swimmers group, 13 in non-swimmers group. (Note: baseline imbalances in months of catheter in place). Effect size not reported.  1.1B. **Total** infections per month in swimmers group: 34 infections per 843 months of catheter in place (0.04%). In non-swimmers group, 13 infections were reported in 506 months of catheter in place (0.025%). RR 1.6, not significant (p-value not reported)  1.1B2. **Tunnel or exit** infections per month in the swimmers group: 20 infections per 843 months of catheter in place (0.02%). In non-swimmers group, 8 infections were reported in 506 months of catheter in place (0.016%). RR 1.5, not significant (p-value not reported)  1.1B3. **Bloodstream infections** per month in the swimmers group: 14 infections per 843 months of catheter in place (0.016%). In non-swimmers group, 5 infections were reported in 506 months of catheter in place (0.009%). RR 1.7, not significant (p-value not reported)  1.2. Number of infections “Frequent swimming” 1.2A. 19 catheter-related infections in frequent swimmers; 27 among infrequent/non-swimmers (Note: baseline imbalances in months of catheter in place). Effect size not reported.  1.2B1. **Total** infections per month in swimmers group: 19 infections per 579 months of catheter in place (0.03%). In infrequent/non-swimmers, 27 infections were reported in 770 months of catheter in place (0.03%). RR 0.9, not significant (p-value not reported) 1.2B2. **Tunnel or exit** infections per month in the swimmers group: 6 infections per 579 months of catheter in place (0.01%). In infrequent/non-swimmers 16 infections were reported in 770 months of catheter in place (0.02%).  1.2B3. **Bloodstream infections** per month in the swimmers group: 13 infections per 579 months of catheter in place (0.02%). In infrequent/non-swimmers, 11 infections were reported in 770 months of catheter in place (0.014%). RR 0.5, not significant (p-value not reported) |  |
|  | Is there important uncertainty about how much people value the main outcomes? | ☐ Important uncertainty or variability  ☐ Possibly important uncertainty or variability  ☐ Probably no important uncertainty or variability  ■ **No important uncertainty or variability**  ☐ No known undesirable outcomes |  |  |
|  | Are the desirable anticipated effects large? | ☐ No  ☐ Probably no  ☐ Uncertain  ☐ Probably yes  ■ **Yes**  ☐ Varies |  |  |
|  | Are the undesirable anticipated effects small? | ☐ No  ■ **Probably no**  ☐ Uncertain  ☐ Probably yes  ☐ Yes  ☐ Varies |  |  |
|  | Are the desirable effects large relative to undesirable effects? | ☐ No  ☐ Probably no  ☐Uncertain  ■ **Probably yes**  ☐ Yes  ☐ Varies |  |  |
| RESOURCE USE | Are the resources required small? | ☐ No  ☐ Probably no  ☐ Uncertain  ☐ Probably yes  ■ **Yes**  ☐ Varies |  |  |
|  | Is the incremental cost small relative to the net benefits? | ☐ No  ☐ Probably no  ☐ Uncertain  ☐ Probably yes  ■ **Yes**  ☐ Varies |  |  |
| EQUITY | What would be the impact on health inequities? | ☐ Increased  ☐ Probably increased  ■ **Uncertain**  ☐ Probably reduced  ☐ Reduced  ☐ Varies |  |  |
| ACCEPTABILITY | Is the option acceptable to key stakeholders? | ☐ No  ☐ Probably no  ☐ Uncertain  ■ **Probably yes**  ☐ Yes  ☐ Varies |  |  |
| FEASIBILITY | Is the option feasible to implement? | ☐ No  ☐ Probably no  ☐ Uncertain  ■**Probably yes**  ☐Yes  ☐ Varies |  |  |

**Conclusion EtD:**

| PROBLEM |  |
| --- | --- |
| Is the problem a priority? | Yes |
| BENEFITS AND HARMS |  |
| What is the overall certainty of this evidence? | Very low quality |
| Is there important uncertainty about how much people value the main outcomes? | No important uncertainty or variability |
| Are the desirable anticipated effects large? | Yes |
| Are the undesirable anticipated effects small? | Probably no |
| Are the desirable effects large relative to undesirable effects? | Probably yes |
| RESOURCE USE |  |
| Are the resources required small? | Yes |
| Is the incremental cost small relative to the net benefits? | Yes |
| EQUITY |  |
| What would be the impact on health inequities? | Uncertain |
| ACCEPTABILITY |  |
| Is the option acceptable to key stakeholders? | Probably yes |
| FEASABILITY |  |
| Is the option feasible to implement? | Probably yes |

**Overall conclusions:**

| Balance of consequences | | | | |
| --- | --- | --- | --- | --- |
| Undesirable consequences  *clearly outweigh*  desirable consequences in most settings  ☐ | Undesirable consequences *probably outweigh*  desirable consequences in most settings  ☐ | The balance between  desirable and undesirable consequences  *is closely balanced or uncertain*  ☐ | **Desirable consequences  *probably outweigh* undesirable consequences in most settings**  ■ | Desirable consequences  *clearly outweigh*  undesirable consequences in most settings  ☐ |

**Recommendation:**

| Type of recommendation – Swimming | | | |
| --- | --- | --- | --- |
| We recommend against  offering this option  ☐ | We suggest not offering  this option  ☐ | **We suggest offering**  **this option**  ■ | We recommend offering  this option  ☐ |
| Recommendation (text) | **We suggest allowing children with cancer to swim (irrespective of neutropenia). (WEAK recommendation, VERY LOW quality of evidence)**  **We strongly believe children with cancer with a non-tunneled central venous catheter such as PICC line should not swim. (STRONG recommendation, EXPERT evidence)** | | |
| Justification | One study in pediatric oncology patients was included for this clinical question. In conclusion, in one study (12), no significant difference in prevalence of infections in the swimmers group versus the non-swimmers group and in the frequent swimmers group versus infrequent/non-swimmers group were reported. This study is small and retrospective, but it does show a trend regarding the effects of swimming, which is supported by our expert opinions.  The guideline panel feels that an absolute restriction regarding swimming is not necessary. If basic hygiene measures are taken into consideration, we feel that children should be allowed to swim in chlorinated water, open water or the sea. We believe not allowing the children to swim, would decrease their quality of life, for example in summer holidays. The panel outweighed the benefits against the harms.   *1. Internal and external tunneled central venous catheters* We believe that children with an internal central venous catheter or an external tunneled central venous catheter, provided that the insertion site and dressings can be cleaned thoroughly should be allowed to swim.  The guideline panel feels that swimming with an internal venous catheter is allowed and would have minimal risks, both infectious and dislocation wise. A specific condition is that the child has an unwounded skin, so no needle should be inserted in the central venous access port or any other type of wound. This would create a potential entry port for bacteria or other and would increase the risk of infection.  The guideline panel agreed that swimming with an external tunneled central venous catheter is allowed and would have minimal risks, both infectious and dislocation wise. A specific condition is that the insertion site of the external venous catheter needs to be thoroughly dried and clean and dry bandages have to be applied after swimming. The guideline panel recognizes the fear for dislocation or problems with the external line from parents and children. Although not necessary, a suggestion is that the child can wear a wetsuit shirt (or a different type of tight shirt) so that the line is pushed against the body.   *2. Non-tunneled lines* There is no evidence for swimming with non-tunneled line. The guideline panel believes that swimming with a non-tunneled line such as a peripheral inserted central catheter (PICC) line, should not be allowed. The guideline panel feels that there is an increased infection risk for non-tunneled lines.   *3. Swimming location*  The guideline panel believes that swimming should be possible in all locations which are destined as swimming areas. For example, chlorinated water (and also swimming lessons), the sea, or in open water, given that there is no general advice against this from the local authorities. We feel that there is minimal difference in infectious risk between chlorinated water, open water and the sea.   In summary, the guideline panel recognizes that the risk of infection or dislocation of the line may be a concern to parents. However, we agree that swimming or swimming lessons increases the quality of life of these children in such a way, that it outweighs the harms. Therefore, we believe children with cancer should be able to swim under certain conditions as the risk of infection would be minimal and the quality of life would decrease with any form of restriction. | | |
| Subgroup considerations | As stated above: there is no evidence for swimming with non-tunneled line. The guideline panel believes that swimming with a non-tunneled line such as a peripheral inserted central catheter (PICC) line, should not be allowed. The guideline panel feels that there is an increased infection risk for non-tunneled lines. | | |
| Implementation considerations | It should be closely monitored by caregivers if children that went swimming get a line infection. It should be noted for every child that gets a line infection. | | |
| Monitoring and evaluation | It should be closely monitored by caregivers if children that went swimming get a line infection. It should be noted for every child that gets a line infection. | | |
| Research priorities | The guideline panel feels that swimming is very important for children with cancer and increases their quality of life. For us, this is a research priority in the upcoming years to make sure children with cancer can swim safely. | | |

**Evidence to decision framework 14: Travelling abroad**

|  | Criteria | Judgements | Research evidence | Additional considerations |
| --- | --- | --- | --- | --- |
| PROBLEM | Is the problem a priority? | ☐ No  ☐ Probably no  ☐ Uncertain  ☐ Probably yes  ■ **Yes** | Improving quality of life has become increasingly important in care for children with cancer. Due to improved survival rates there is an increased focus on morbidity and adverse effects of anti-cancer treatment (1, 2). To prevent adverse health problems, such as infections and bleeding, restrictions in daily life have been defined for children with cancer related to school attendance, travelling with public transport, pets, hygiene measures and swimming (3). However, these social restrictions can severely impair the quality of life of these children (4, 5).  Within the Netherlands, there is large variation in current supportive care practices, including social restrictions (6). The majority of these recommendations regarding social restrictions for children with cancer are not evidence-based. Guidance is necessary in order to provide the best possible care for these children, balancing cautiousness and restrictiveness. |  |
| BENEFITS AND HARMS | What is the overall certainty of this evidence? | ■ **No included studies**  ☐ Very low  ☐ Low  ☐ Moderate  ☐ High | No evidence in pediatric oncology patients was found for this clinical question. Therefore, the recommendation is based on expert opinions.  The guideline panel believes that children with cancer can travel abroad, provided that they visit a country with a comparable health system and provided that the child is in good clinical health. We see no obvious reasons why this should be contraindicated in stable children.  However, this should always be a careful consideration for the child as an individual, and therefore this always needs to be discussed and be allowed by the treating physician. It should not interfere with treatment and parents should carry a letter of the treating physician, in the event something happens when abroad. |  |
|  | Is there important uncertainty about how much people value the main outcomes? | ☐ Important uncertainty or variability  ☐ Possibly important uncertainty or variability  ☐ Probably no important uncertainty or variability  ■ **No important uncertainty or variability**  ☐ No known undesirable outcomes |  |  |
|  | Are the desirable anticipated effects large? | ☐ No  ☐ Probably no  ☐ Uncertain  ■ **Probably yes**  ☐ Yes  ☐ Varies |  |  |
|  | Are the undesirable anticipated effects small? | ☐ No  ☐ Probably no  ☐ Uncertain  ■**Probably yes**  ☐ Yes  ☐ Varies |  |  |
|  | Are the desirable effects large relative to undesirable effects? | ☐ No  ☐ Probably no  ☐Uncertain  ■ **Probably yes**  ☐ Yes  ☐ Varies |  |  |
| RESOURCE USE | Are the resources required small? | ☐ No  ☐ Probably no  ☐ Uncertain  ■ **Probably yes**  ☐ Yes  ☐ Varies |  | Not from the healthcare perspective. |
|  | Is the incremental cost small relative to the net benefits? | ☐ No  ☐ Probably no  ☐ Uncertain  ■ **Probably yes**  ☐ Yes  ☐ Varies |  |  |
| EQUITY | What would be the impact on health inequities? | ☐ Increased  ☐ Probably increased  ■ **Uncertain**  ☐ Probably reduced  ☐ Reduced  ☐ Varies |  | The panel expects that this daily life restriction will not have any effect on health inequities. |
| ACCEPTABILITY | Is the option acceptable to key stakeholders? | ☐ No  ☐ Probably no  ☐ Uncertain  ■ **Probably yes**  ☐ Yes  ☐ Varies |  |  |
| FEASIBILITY | Is the option feasible to implement? | ☐ No  ☐ Probably no  ☐ Uncertain  ■ **Probably yes**  ☐Yes  ☐ Varies |  |  |

**Conclusion EtD**:

| PROBLEM |  |
| --- | --- |
| Is the problem a priority? | Yes |
| BENEFITS AND HARMS |  |
| What is the overall certainty of this evidence? | No included studies |
| Is there important uncertainty about how much people value the main outcomes? | No important uncertainty or variability |
| Are the desirable anticipated effects large? | Probably yes |
| Are the undesirable anticipated effects small? | Probably yes |
| Are the desirable effects large relative to undesirable effects? | Probably yes |
| RESOURCE USE |  |
| Are the resources required small? | Probably yes |
| Is the incremental cost small relative to the net benefits? | Probably yes |
| EQUITY |  |
| What would be the impact on health inequities? | Uncertain |
| ACCEPTABILITY |  |
| Is the option acceptable to key stakeholders? | Probably yes |
| FEASABILITY |  |
| Is the option feasible to implement? | Probably yes |

**Overall conclusions:**

| Balance of consequences | | | | |
| --- | --- | --- | --- | --- |
| Undesirable consequences  *clearly outweigh*  desirable consequences in most settings  ☐ | Undesirable consequences *probably outweigh*  desirable consequences in most settings  ☐ | The balance between  desirable and undesirable consequences  *is closely balanced or uncertain*  ☐ | Desirable consequences  *probably outweigh* undesirable consequences in most settings  ☐ | **Desirable consequences  *clearly outweigh*  undesirable consequences in most settings**  ■ |

**Recommendation:**

| Type of recommendation – Travelling abroad | | | |
| --- | --- | --- | --- |
| We strongly believe not to  ☐ | We believe not to  ☐ | We believe that  ☐ | **We strongly believe that**  ■ |
| Recommendation (text) | **We strongly believe that children with cancer can travel abroad, provided that they visit a country with a comparable health system and provided that the child is in good clinical health. (STRONG recommendation, EXPERT evidence)** | | |
| Justification | No evidence in pediatric oncology patients was found for this clinical question. Therefore, the recommendation is based on expert opinions.  The guideline panel believes that children with cancer can travel abroad, provided that they visit a country with a comparable health system and provided that the child is in good clinical health. We see no obvious reasons why this should be contraindicated in stable children. | | |
| Subgroup considerations | This recommendation accounts for clinically stable children. | | |
| Implementation considerations | This should always be a careful consideration for the child as an individual, and therefore this always needs to be discussed and be allowed by the treating physician. It should not interfere with treatment and parents should carry a letter of the treating physician, in the event something happens when abroad. | | |
| Monitoring and evaluation | Not applicable. | | |
| Research priorities | Not applicable. | | |
